# CDC25A inhibition sensitizes melanoma cells to doxorubicin and NK cell therapy

**DOI:** 10.1038/s41419-025-07598-w

**Published:** 2025-04-11

**Authors:** Xinyue Gao, Feichang Liu, Bo Zhang, Tianyi Ren, You Zheng, Zubiao Niu, He Ren, Chenyu Liu, Chengzuo Jiang, Chenxi Wang, Hongyan Huang, Li Ma, Qiang Sun

**Affiliations:** 1 Laboratory of Advanced Biotechnology, Beijing Institute of Biotechnology; Research Unit of Cell Death Mechanism, Chinese Academy of Medical Science, 2021RU008, Beijing, China; 2 Institute of Molecular Immunology, School of Laboratory Medicine and Biotechnology, Southern Medical University, Guangzhou, China; 3 Department of Oncology, Beijing Shijitan Hospital, Capital Medical University, Beijing, China; 4 Department of Interventional Pulmonology, Affiliated Cancer Hospital of Zhengzhou University & Henan Cancer Hospital, Zhengzhou, China; 5 Department of Biology, Hainan Medical University, Haikou, China

**Keywords:** Cancer therapeutic resistance, Cell death, Melanoma

## Abstract

Cell division cycle 25 (CDC25) phosphatases serve as crucial regulators of cell cycle phase transitions and essential components of the checkpoint machinery involved in DNA damage response. Emerging evidence indicates the oncogenic potential of CDC25 family members across various cancers. However, comprehensive insights into the expression pattern and function of the CDC25 family in diverse cancers remain unexplored. In our study, we investigated CDC25 family using multiple databases, including gene expression levels, molecular signatures, diagnosis value, and prognostic value in pan-cancer. Furthermore, we focused on melanoma and systematically explored CDC25A expression and its clinical correlations. As a result, the expression of CDC25 family members is significantly abnormal in most cancers, correlating with poorer prognosis. CDC25 family members are differently regulated by DNA methylation and genetic alterations across various cancers. In addition, CDC25 family plays a critical role in the malignant progression of melanoma. Functional investigation reveals that CDC25A inhibition suppresses the proliferation of melanoma cells and sensitizes melanoma cells to chemotherapy and NK cell therapy. In conclusion, our study suggests that CDC25 family may serve as a significant biomarker for diagnosis and prognosis across multiple cancers, with CDC25A as a promising therapeutic target for melanoma.

## Introduction

Cancer is one of the leading causes of death worldwide, posing a significant barrier to the improvement of public health [1]. The latest global cancer statistics, published in 2024, report nearly 20 million new cancer cases and 10 million cancer-related deaths in 2022 [2]. Although chemotherapy, targeted therapy and immunotherapy have shown remarkable advancements in recent decades, substantial challenges persist in cancer treatment, including tumor resistance, poor clinical responses, and the absence of reliable biomarkers [3, 4]. The molecular complexity of melanoma, from driver mutations to dynamic phenotype switching, makes it a model for understanding cancer evolution and therapy resistance [5, 6]. Over the past decades, novel therapeutic strategies including immunotherapy, targeted therapy, and combination therapy are emerging, which have led to great improvements in the survival of advanced patients [7, 8]. The absence of early diagnostic biomarkers and therapeutic limitations such as off-target effects remain major clinical challenges in the contemporary treatment of melanoma. Therefore, identifying novel biomarkers that can accurately predict treatment efficacy has become crucial.

Aberrant cell cycle progression is a key mechanism in tumorigenesis [9]. In tumors, it typically drives continuous and excessive cell division, thereby promoting tumor progression [10]. Consequently, key regulators of the cell cycle may serve as efficient targets for anti-tumor therapies. Cell division cycle 25 (CDC25) dual phosphatases are essential components of cell cycle control which activate cyclin-dependent kinases (CDKs) through dephosphorylation, thereby promoting cell cycle progression. The abnormal expression and distribution of CDC25 family members are implicated in cancer initiation, progression, and poor prognosis. In mammalian cells, the CDC25 phosphatase family consists of three members: CDC25A, CDC25B, and CDC25C [11]. Compared to the highly conserved catalytic domain, the regulatory domains of the three members exhibit greater diversity, leading to distinct subcellular localization and function [12]. The primary function of CDC25A pertains to the cell cycle, with its involvement spanning the entire process. Unlike CDC25B and CDC25C, CDC25A facilitates the G1/S phase transition [13]. In addition to its critical role in cell cycle regulation, CDC25A assumes a pivotal part in biological processes including DNA damage repair, apoptosis, and cellular senescence [14–16]. CDC25A represents a potential protein that promotes tumor progression through cell cycle regulation. It has been identified as a biomarker for predicting prognosis in numerous tumor types [17, 18]. CDC25B and CDC25C both promote the G2/M phase transition via the activation of CDK1. CDC25B initiates the transition of cells from the G2 to the M phase and is essential for cell cycle progression [12]. Additionally, CDC25B can enhance the activation of estrogen receptor, androgen receptor (AR), and progesterone receptor, thereby promoting tumor malignancy [19]. High expression of CDC25B exhibits a positive correlation with poor prognosis in esophageal squamous cell carcinoma, pancreatic ductal adenocarcinoma, and non-small cell lung carcinoma [19]. CDC25C is responsible for promoting and sustaining the complete activation of cyclin B1/CDK1, ultimately ensuring the smooth transition of cells through the G2 checkpoint [13]. Overexpression, alternative splicing, and phosphorylation of CDC25C are intricately linked to tumorigenesis and the formation of polyploid giant cancer cells [20–22]. In addition, CDC25C may be involved in energy metabolism by maintaining mitochondrial homeostasis [23]. Previous studies have confirmed the vital roles of CDC25 family members in different tumor development and treatment, however, a comprehensive pan-cancer analysis of the CDC25 family has yet to be conducted.

In this study, we comprehensively analyzed the expression patterns, genetic and epigenetic alterations, and clinical significance of CDC25 family members in pan-cancer via various databases. Our results indicated that the expression of CDC25 family was aberrant across most cancers, associating with a poor prognosis. In most cancers, diverse genetic mutations, and epigenetic alterations of CDC25 family were observed. Furthermore, we selected melanoma as a representative cancer to confirm the expression and function of CDC25 family. Our results indicated that CDC25 family members, particularly CDC25A, play a critical role in the malignant progression of melanoma. We found that CDC25A inhibition could suppress the proliferation of melanoma cells and sensitize melanoma cells to doxorubicin and natural killer (NK) cell therapy. Together, our study illustrates that CDC25 family members are potential biomarkers for prognosis in pan-cancer and promising therapeutic targets for melanoma.

## Results

### Abnormal expression of the CDC25 family in tumor tissues

**Fig. 1:**
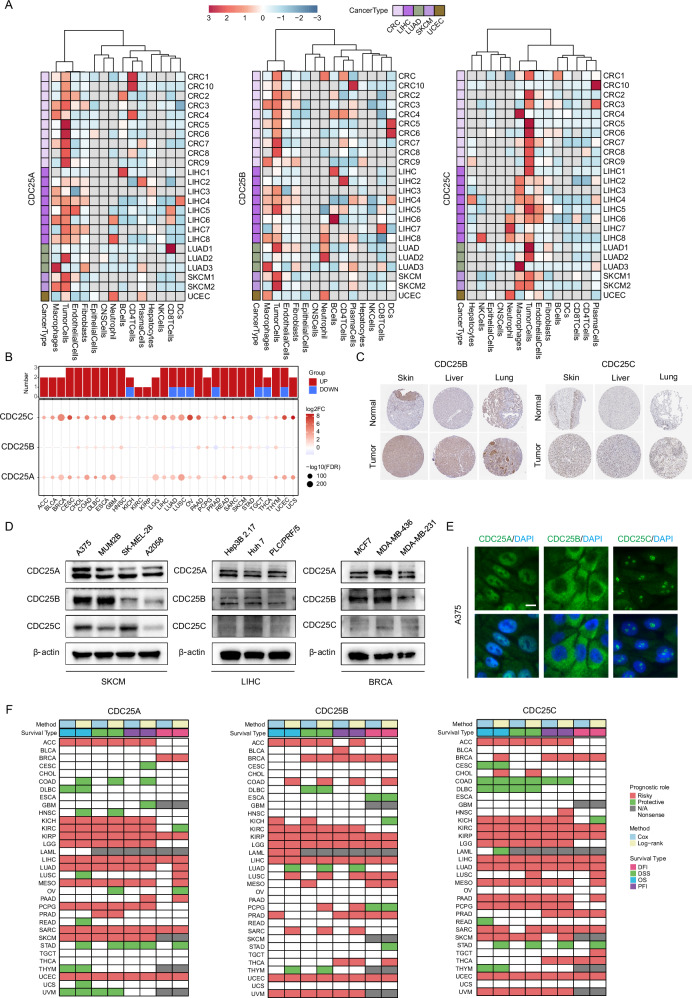
Differential expression of CDC25 family genes across various cancers. **A** The heatmap illustrates the average expression of genes across each cell type within each slice at the spatial transcriptomic level. **B** Differential expression of genes between tumor and normal tissues across a pan-cancer cohort. **C** Immunohistochemical images results of CDC25 family members in normal and corresponding cancer tissues, as provided by the HPA database. **D** Differential expression of CDC25 family members in breast cancer, liver cancer, and melanoma cell lines. **E** Immunofluorescence images depicting CDC25 family members in melanoma cell line. Scale bars: 10 μm. **F** Survival prognosis analysis of cancers with CDC25 family members expression in Kaplan–Meier and Cox proportional hazard regression analysis.

To investigate the expression pattern of the CDC25 family, in tumor and normal tissues, we first utilized various spatial transcriptome datasets. We observed enhanced expression of the CDC25 family in malignant cell microregions (Fig. 1A). Similar results were also displayed in single-cell transcriptome datasets (Fig. S1).

Additionally, the TCGA and GTEx data were integrated to examine the expression patterns across various cancers. The result showed that expression of CDC25 family was consistently upregulated in 14 types of cancer (cervical squamous cell carcinoma and endocervical adenocarcinoma (CSEC), cholangiocarcinoma (CHOL), colon adenocarcinoma (COAD), lymphoid neoplasm diffuse large B-cell lymphoma (DLBC), esophageal carcinoma (ESCA), glioblastoma multiforme (GBM), head and neck squamous cell carcinoma (HNSC), liver hepatocellular carcinoma (LIHC), pancreatic adenocarcinoma (PAAD), rectum adenocarcinoma (READ), sarcoma (SARC), skin cutaneous melanoma (SKCM), stomach adenocarcinoma (STAD), and thymoma (THYM)) while downregulated in certain cancers. For instance, CDC25A expression was downregulated in kidney renal clear cell carcinoma (KIRC), CDC25B was downregulated in kidney chromophobe (KICH), lung adenocarcinoma (LUAD), lung squamous cell carcinoma (LUSC), ovarian serous cystadenocarcinoma (OV), prostate adenocarcinoma (PRAD), thyroid carcinoma (THCA), and uterine corpus endometrial carcinoma (UCEC), and CDC25C was downregulated in testicular germ cell tumors (TGCT) (Fig. 1B).

To further investigate protein levels of CDC25 family, immunohistochemistry data were obtained from The Human Protein Atlas (HPA) database (CDC25A data were unavailable). As shown in Figs. 1C and S2A, CDC25B/C exhibited strong staining in cancer tissues, indicating high expression. Next, cell lines of LIHC, breast cancer (BRCA), and SKCM were assessed for CDC25 family members' expression by Western blotting, revealing substantial and differential expression across different cell lines (Fig. 1D). In addition, we assessed the expression of CDC25 family members at the subcellular level using immunofluorescence staining. CDC25A/C were predominantly expressed in the nucleus, whereas CDC25B was expressed in the cytoplasm (Fig. 1E). In summary, these findings suggest that CDC25 family members are proficiently expressed in cancers.

### Prognostic and predictive value of CDC25 family across various cancers

To explore the prognostic values, CDC25 family members were evaluated for their relationships with overall survival (OS) (or disease-free survival (DSS), progression-free interval (PFI), disease-free interval (DFI)) across pan-cancer types using the TCGA dataset (Fig. 1F). Our analysis revealed that CDC25 family members acted as patient risk factors in the TCGA cohort, that is, patients with high expression of CDC25 family members exhibited poorer survival in 11 cancer types (adrenocortical carcinoma (ACC), BRCA, KICH, KIRC, kidney renal papillary cell carcinoma (KIRP), brain lower grade glioma (LGG), LIHC, mesothelioma (MESO), PRAD, SARC, and UCEC) (hazard ratio (HR) > 1, *p* < 0.05). Whereas in several tumors, such as DLBC and STAD, CDC25 family members acted as a protective factor (HR < 1, *p* < 0.05). Meanwhile, this conclusion was validated using external datasets, that is, high CDC25 family expression was associated with poor prognosis in cancer patients. Notably, in STAD patients, elevated CDC25 family expression was associated with prolonged survival. Additionally, the ROC curve demonstrated that CDC25 family could universally discriminate between cancer patients and non-cancer controls across 12 cancer types (Fig. S2B, area under the curve (AUC) > 0.7) with high diagnostic efficiency in COAD (AUC > 0.9). Overall, our findings suggest that CDC25 family serves as an independent predictive factor for multiple cancers.

### DNA methylation and genetic alterations of CDC25 family members

**Fig. 2:**
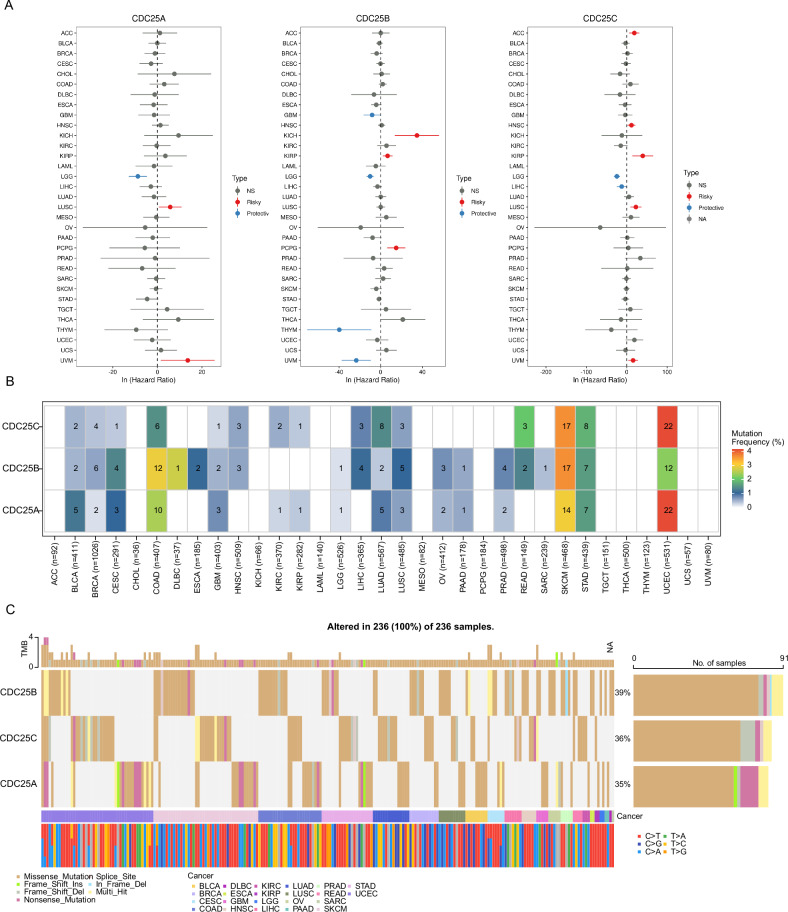
Genetic alteration features of CDC25 family members in pan-cancer. **A** Survival prognosis analysis of cancers based on CDC25 methylation levels using Cox proportional hazard regression analysis. **B**, **C** Frequency and types of mutation in CDC25 family members.

Aberrant DNA methylation had been reported to contribute to tumor progression, accordingly, the promoter DNA methylation levels of CDC25 family members were analyzed using the UCSC Xena database, which revealed a common alteration in three cancer types: KIRP, LUSC, and PRAD (Fig. S3, *p* < 0.05). In detail, CDC25A was significantly methylated in 5 types of tumors, including BRCA, KIRC, KIRP, PRAD, and UCEC, while it was hypomethylated in COAD and LUSC. The methylation level of CDC25B was upregulated in KIRC, KIRP, LUSC, PRAD, and HNSC but downregulated in LIHC. Distinct from CDC25A and CDC25B, the methylation level of CDC25C decreased in most tumor tissues, including KIRP, LUSC, PRAD, UCEC, LIHC, BLCA, LUAD, and pheochromocytoma and paraganglioma (PCPG). Furthermore, the correlation between CDC25 family members' methylation and OS was explored by Cox regression analysis across various cancer types, indicating high heterogeneity across different tumors (Fig. 2A). CDC25 family members were associated with OS (*p* < 0.05) and were identified as potential risk factors (HR > 1). In LGG, CDC25 family members were correlated with patient survival and were protective factors (HR < 1, *p* < 0.05).

Next, the mutational profile of CDC25 family members were analyzed in human cancers using the cBioProtal database based on TCGA datasets. As shown in Fig. 2B, C, ~3–4% of patients experienced CDC25 family mutations in CESC, COAD, LUAD, SKCM, STAD, and UCEC, with the predominant types being missense mutation. In detail, CDC25C was most frequently altered in UCEC, with more than 4% of patients exhibiting mutations, followed by SKCM, where 3% of patients showed amplification. High mutation frequencies also occurred in COAD and SKCM, with ~3% of cases involving mutations in CDC25B.

### CDC25 family expression is associated with malignancy in melanoma

Next, melanoma was selected as a representative cancer to explore CDC25 family members’ pathological functions in human cancer based on the above pan-cancer analysis that, in melanoma, CDC25 family members were significantly upregulated, associated with poor prognosis, and displayed a higher mutational frequency.

**Fig. 3:**
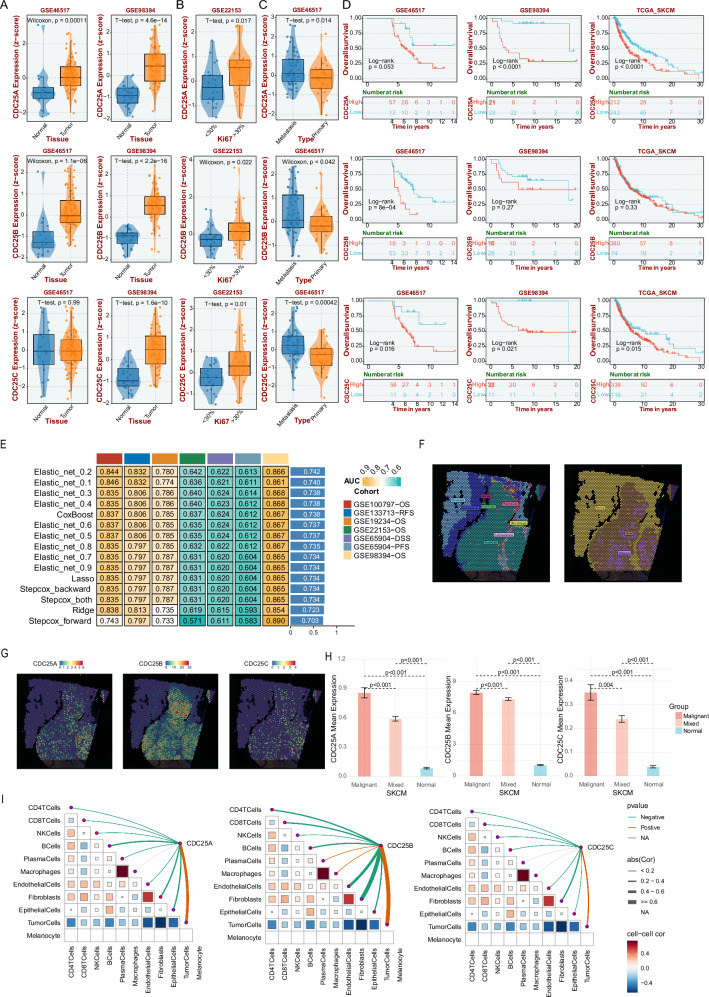
The expression and clinical association analysis of CDC25 in SKCM tumor tissue. **A** Differential expression of CDC25 family members between the GSE46517 and GSE98394 cohorts. **B** Differential expression of CDC25 family members between the Ki67++ and Ki67− groups in the GSE22153 cohort. **C** Differential expression of CDC25 family members between the primary and metastatic groups in the GSE46517 cohort. **D** Survival prognosis analysis of cancer with CDC25 expression in Kaplan–Meier analysis. **E** Comparison of mean AUC values at 1, 3, and 5 years for prognostic models constructed by different algorithms. This heatmap shows the average AUC values for each time point. **F** Deconvolution analysis was employed to accurately assess cell composition at each point on the 10x Visium slide. Each dot represents a microregion (spot) sequenced by spatial transcriptomics, with darker red indicating a higher cell count. **G** Based on deconvolution results, the cell type with the highest composition in each microregion is calculated and visualized. Each dot represents a spot from spatial transcriptome sequencing, with different colors denoting different cell types. **H** Wilcoxon rank-sum tests were used to evaluate the statistical significance of gene expression differences between the three groups. **I** Spearman correlation analysis was performed to assess the correlation between cell counts and gene expression in all spots. The red line represents a positive correlation, the green line a negative correlation, and the gray line indicates non-significance. The thickness of the line corresponds to the absolute value of the correlation coefficient. In the triangular region, the color depth and square size represent the correlation strength, with red indicating positive correlation and blue indicating negative correlation. The darker the color and the larger the square, the more significant the correlation.

First, we further investigated the CDC25 family members' expression in melanoma and non-tumor tissues using independent cohorts. RNA-seq data confirmed that CDC25 family expression was elevated in tumor tissues (GSE46517 and GSE98394, Fig. 3A). And high CDC25 family expression was identified in Ki67++ tumor and metastasis sites (Fig. 3B, C), suggesting a crucial role in the proliferation and metastasis of melanoma, though the mRNA level of CDC25 family members was not correlated with pathological grade (Fig. S4A, B). Then, the best-performing thresholds were used to set cutoffs and displayed the Kaplan–Meier plots of OS, DSS and PFS in three datasets. As a result, high expression of CDC25A/C, but not CDC25B, predicted short survival of melanoma patients in OS, DSS, or PFS (Figs. 3D and S4C–E). Consistently, univariate, and multivariate regression analyses also showed that overexpression of CDC25A and CDC25C, but not CDC25B, was correlated with unfavorable OS in TCGA-SKCM datasets (Fig. S4A, HR > 1, *p* < 0.05). Furthermore, various algorithms were employed to construct melanoma patients’ prognostic models to comprehensively evaluate the average AUC values of 1, 3, and 5 years, with the Elastic_net model being the best algorithm. The conclusion was reached through an exhaustive comparative analysis, with the Elastic_net_0.2 model standing out for its excellent performance in the average AUC values at all three time points (Fig. 3E).

Two spatial transcriptome datasets were analyzed to define the differences in the spatial distribution of CDC25 family members. To accurately assess the cell composition at each point on the 10x Visium slide, deconvolution analysis was applied. Based on the above results, we calculated the cell type with the highest content of each microregion and visualized the maximum cell composition of each microregion (Figs. 3F and S4F, G). The high expression regions of CDC25 family members were highly coincident in melanoma, primarily in malignant regions. (Fig. 3G), followed by mixed malignant regions, and the lowest in normal regions (Figs. 3H and S4H). Consistently, the expression of CDC25 family members was positively correlated with the content of tumor cells at each spot (Figs. 3I and S4I). In order to gain a comprehensive understanding of the function characteristics of CDC25 family members in melanoma, the Pearson correlation of GSVA scores between *z*-scores of CDC25 family members' expression level and 14 cancer functional states was analyzed. In melanoma, the expression of CDC25A was positively correlated with cell cycle, DNA damage, DNA repair, and proliferation scores, and negatively correlated with angiogenesis, differentiation, inflammation, and quiescence scores (Fig. S5A). The expression of CDC25B was positively correlated with cell cycle scores, and negatively correlated with angiogenesis, differentiation, epithelial-mesenchymal transition (EMT), invasion, quiescence, and stemness scores (Fig. S5B). In addition, it had a significantly positive correlation between CDC25C expression and apoptosis, cell cycle, DNA damage, DNA repair, EMT, invasion, proliferation, and stemness scores (Fig. S5C). Based on CDC25A expression levels, melanoma patients were divided into quartiles (Q1–Q4), with Q1 denoting the highest-expressing 25% of samples and Q4 representing the lowest-expressing 25%. Quantitative assessment of key cell cycle-associated genes and the EMT-associated genes was performed across the quartile groups. The Q1 cohort demonstrated significantly elevated expression of CCNA2, CCNB1, CCND1, and CDH2, indicating CDC25A’s potential regulatory role in coordinating melanoma cell cycle progression with EMT dynamics. (Fig. S5D, E). Above all, these results indicate that CDC25 family members are upregulated in melanoma, associated with poor prognosis, tumor proliferation, and metastasis, making them critical players in melanoma progression.

### DNA methylation features of CDC25 family members in melanoma

**Fig. 4:**
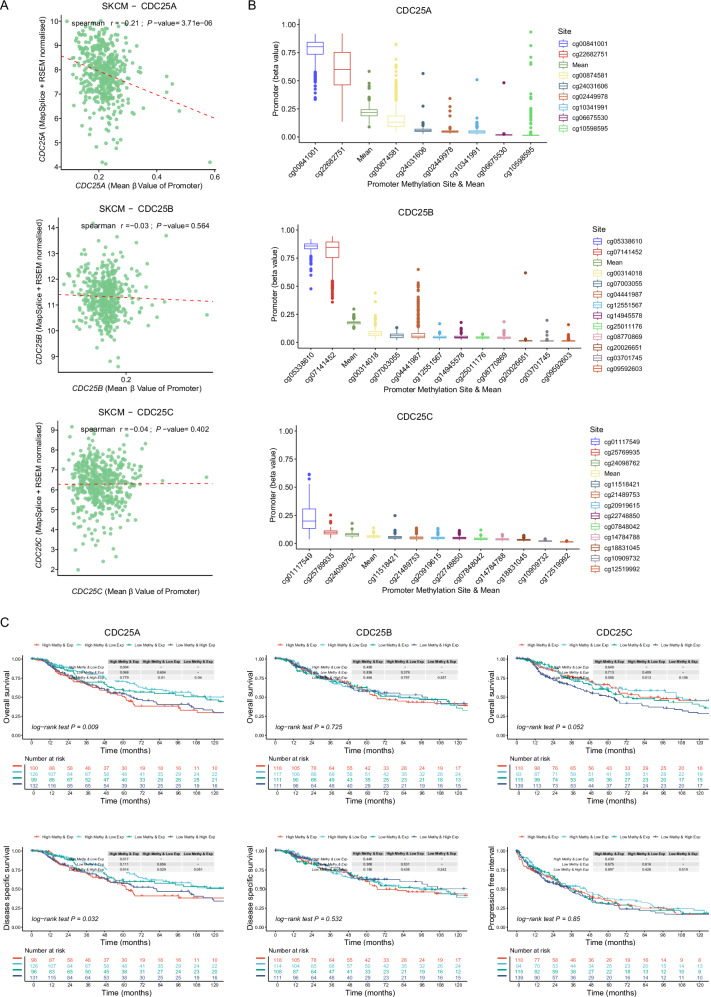
The promoter methylation features of CDC25 family members in SKCM. **A** Spearman correlation between the methylation level of CDC25 family members and its mRNA expression in SKCM. Each scatter represents a sample. **B** The methylation levels at different sites of CDC25 family members promoter were sequenced. Each scatter represents a different sample. **C** Kaplan–Meier survival analysis for OS and DSS of SKCM patients based on CDC25 family members expression and methylation. The expression levels of CDC25 family members are standardized using a *z*-score with respect to the methylation degree in specific regions. A *z*-score being greater than 0, defined as high expression or hypermethylated groups, otherwise as low expression or hypomethylated groups.

To investigate whether the expression pattern of CDC25 family members in melanoma is related to gene methylation, spearman correlation analysis was applied. The results demonstrated a significantly negative correlation between the expression and methylation level of CDC25A, whereas no significant association was observed between CDC25B/C expression and their respective methylation levels (Fig. 4A). Next, the visualization of CDC25 family members promoter methylation level revealed that CDC25A promoter region contained 8 methylation sites, whereas CDC25B and CDC25C promoters each had 12 sites. Specifically, sites cg00841001 and cg22682751 (CDC25A), cg05338610 and cg07141452 (CDC25B), and cg01117549 (CDC25C) exhibited significantly elevated methylation levels, suggesting that CDC25 family members are potential methylation regulated genes (Fig. 4B). To verify the synergistic effect of CDC25 family members expression and methylation levels in the prognosis of melanoma patients, we normalized the expression and methylation levels of CDC25 family members using *z*-scores to obtain four subgroups, followed by Kaplan–Meier survival analysis (Fig. 4C). The high methylation and expression level of CDC25A subgroup patients exhibited worse OS and DSS compared to the other three subgroups. These results indicate that CDC25A methylation has a significant correlation with its expression level and has an impact on the survival of melanoma patients.

### CDC25A inhibition exhibits tumor-suppressive effects

**Fig. 5:**
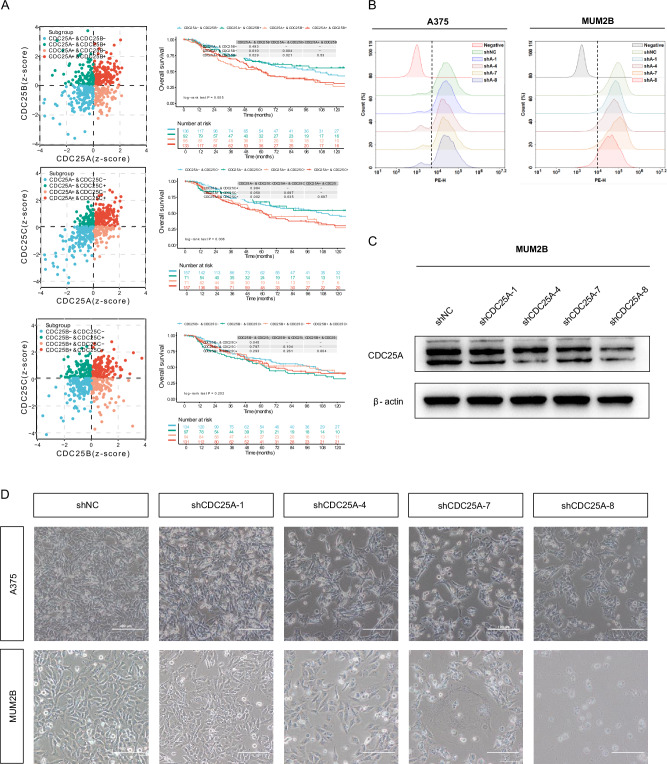
CDC25A plays a crucial role in melanoma cells. **A** The synergistic effects of CDC25 family member expression were analyzed. The left panel shows scatter plots representing the *z*-scores of the samples, where each point represents a sample. Different colors indicate distinct subgroups and the horizontal/vertical axes correspond to the *z*-scores of the two genes, respectively. A *z*-score ≤ 0 indicates low expression, while a *z*-score > 0 indicates high expression. The right panel shows a Kaplan–Meier survival analysis of melanoma patients based on the expression of CDC25 family members. **B** The expression levels of mCherry were analyzed in A375 and MUM2B CDC25A-KD cells by flow cytometry. **C** Western blot assessed the protein level of CDC25A in A375 CDC25A-KD cells. **D** Representative images of A375 and MUM2B CDC25A-KD cells were shown. Scale bars: 100 μm.

To explore the interaction and relationship between CDC25 family members, we normalized the expression levels of each gene pair using *z*-scores to obtain four subgroups (Fig. 5A), followed by Kaplan–Meier survival analysis. Results showed that the CDC25A + &CDC25B+ and CDC25A + &CDC25B− subgroups exhibited worse survival outcomes compared to the CDC25A−&CDC25B+ and CDC25A−&CDC25B− subgroups. The CDC25A + &CDC25C+ and CDC25A + &CDC25C− subgroups also exhibited worse survival compared to the CDC25A−&CDC25C+ and CDC25A−&CDC25C− subgroups. Taken together, our studies demonstrate that, within the CDC25 family, CDC25A, rather than CDC25B or CDC25C, plays a more dominant role in melanoma progression. As a functional validation, four stable CDC25A-knockdown cell lines were constructed in A375 and MUM2B cells (Fig. 5B). Western blot confirmed CDC25A knockdown in three hairpins (Fig. 5C), resulting in a significant change in cellular morphology, appearance of multinucleated cells, and compromised cell proliferation and eventual cell death (Fig. 5D).

**Fig. 6:**
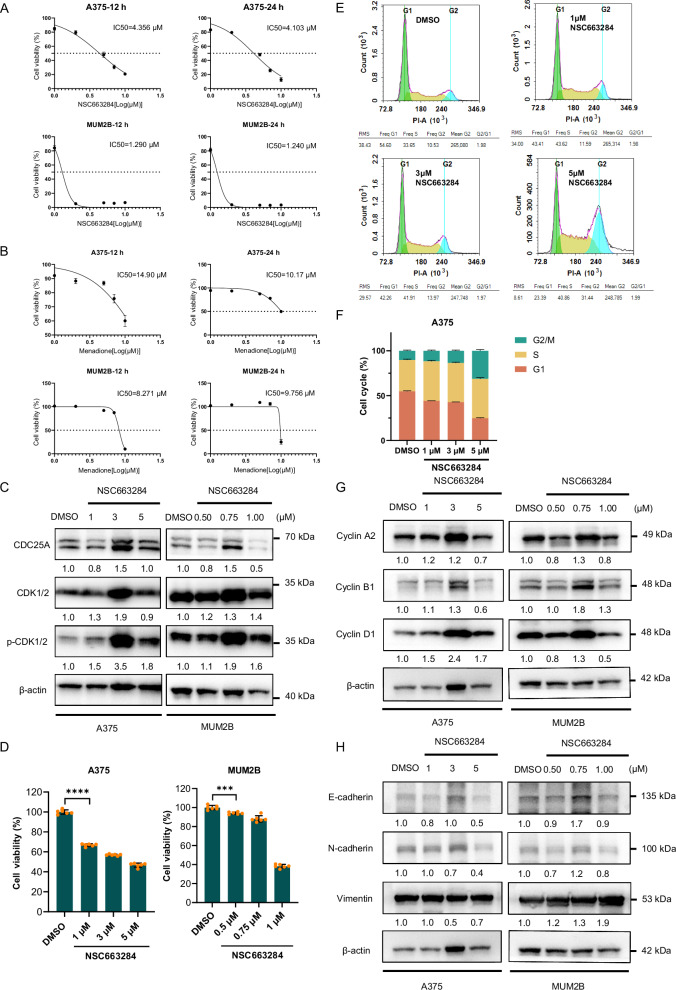
The inhibition of CDC25A suppresses the proliferation of melanoma cells. **A**, **B** Dose-response curves for A375 and MUM2B cells exposed to NSC663284 or menadione for 12 and 24 h. **C** Increased phosphorylation of CDK1/2 was detected by western blot, confirming that NSC663284 effectively inhibited the function of CDC25A phosphatase in A375 and MUM2B cells. The β-actin was used as a loading control. **D** CCK-8 assays demonstrated that NSC663284-induced CDC25A inhibition suppressed proliferation in A375 and MUM2B cells. **E**, **F** Cell cycle assays were performed to evaluate the effects of varying concentrations of NSC663284 on A375 cells. **G** Expression of cell cycle molecules upon CDC25A inhibition detected by western blot. The β-actin was used as a loading control. **H** Expression of EMT-related molecules following CDC25A inhibition was detected by Western blot analysis. The β-actin was used as a loading control.

Given that CDC25 family proteins are phosphatases capable of promoting the cell cycle by dephosphorylating CDK, specific inhibitors, including NSC663284 and menadione, were employed to explore their function in melanoma. A375 and MUM2B cells were treated with the two CDC25A inhibitors. As a result, NSC663284 exhibited a superior inhibitory effect on cell proliferation compared to menadione (Fig. 6A, B). The phospho-CDK1/2 level/total CDK1/2 level was elevated upon NSC663284 treatment, indicating an inhibition of CDC25A phosphatase (Fig. 6C). CCK-8 assays indicated that CDC25A inhibition decreased cell proliferation (Fig. 6D). Similarly, CDC25A inhibition induced cell cycle arrest in G2/M phase in A375 cells (Fig. 6E, F), concomitantly with an increase in the expression of several cell cycle-related genes, including cyclin A2, cyclin B1, and cyclin D1 (Fig. 6G). Also, inhibiting CDC25A suppressed the EMT process of melanoma cells as evidenced by increased expression of E-cadherin, as well as decreased N-cadherin and vimentin expression (Fig. 6H). In conclusion, these results are consistent with a role of CDC25A inhibition in suppressing cell proliferation and disrupting the cell cycle in melanoma cells.

### CDC25A inhibition sensitizes melanoma cells to doxorubicin chemotherapy

**Fig. 7:**
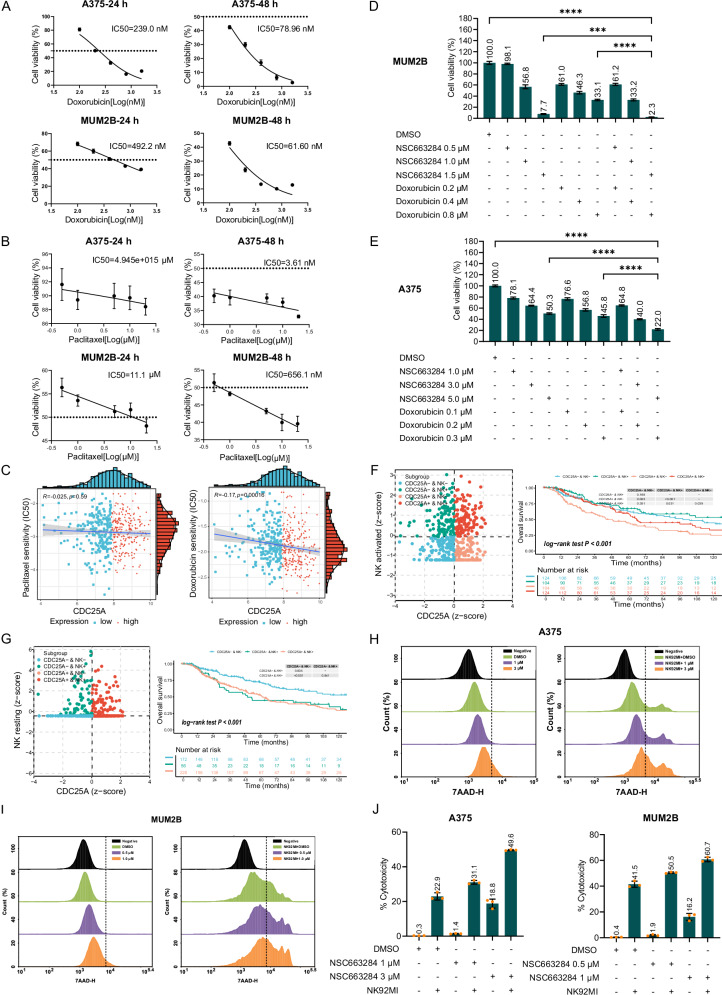
CDC25A inhibition sensitizes melanoma cells to doxorubicin and NK cell killing. **A**, **B** Dose-response curves of A375 and MUM2B cells exposed to Doxorubicin or paclitaxel for 24 and 48 h. **C** Spearman correlation analysis of the relationship between CDC25A expression levels and drug sensitivity (IC50 values). Each scatter point represents a sample. The horizontal and vertical of the scatter point denote the gene expression level and the drug IC50 value of the sample respectively. Samples were classified into high-expression (red) and low-expression (blue) groups based on the median gene expression level. **D**, **E** CCK-8 assays evaluated the susceptibility of NSC663284 and doxorubicin in A375 and MUM2B cells. The synergistic effects of CDC25A expression and activated NK cell (**F**) and resting NK cell (**G**) infiltration on survival were analyzed. The left panels are scatter diagrams of *z*-scores of the samples, each scatter represents a sample, and different colors represent different subgroups. *Z*-scores ≤ 0 represent low expression, while *z*-scores > 0 indicate high expression. The right panels show the survival analysis of melanoma patients in each subgroup in Kaplan–Meier analysis. **H**–**J** CDC25A inhibition sensitized A375 and MUM2B cells to NK cell killing, as shown by 7-AAD staining and CCK-8 assay.

Paclitaxel and doxorubicin are widely used chemotherapeutic agents in the clinical treatment of melanoma. Drug resistance is common during therapy, calling for novel drug recipes. Given the mechanistic difference of CDC25A, we hypothesized an enhanced therapeutic effect for paclitaxel and doxorubicin upon simultaneous CDC25A inhibition. As shown in Fig. 7A–C, doxorubicin and paclitaxel treatment inhibited the growth of A375 and MUM2B cells in a dose- and time-dependent manner (Fig. 7A, B), and correlation analysis indicated that the sensitivity of doxorubicin, but not paclitaxel, was negatively correlated with CDC25A mRNA level (Fig. 7C). As a functional validation, we treated melanoma cells with doxorubicin in combination with NSC663284. The results revealed that cell viability in combination groups was lower than those in control or single-drug groups (Fig. 7D, E). In conclusion, these results suggest that CDC25A inhibition could increase the sensitivity of melanoma to doxorubicin.

### CDC25A inhibition enhances NK cell killing of melanoma cells

The clinical application of targeted and cytotoxic T cell (CTL)-based immune checkpoint therapies has increased the survival rate of melanoma patients over the last decade [24]. NK cells, as the “first responders” in tumor and viral immune defense, can exert their function without prior antigen-specific stimulation [25]. However, NK cells are not yet widely utilized in clinical anti-melanoma therapies. Therefore, we explored the synergistic effects of CDC25A expression and infiltration of activated or resting NK cells on melanoma patient survival. As a result, the subgroup with low CDC25A expression and high infiltration of activated NK cells displayed the best OS among the four subgroups (Fig. 7F). Correspondingly, the patients with high CDC25A expression and high infiltration of resting NK cells had worse OS (Fig. 7G). These data indicated that tumor-infiltrating NK cells strongly affected the OS of melanoma patients. To further validate the role of CDC25A in the melanoma sensitivity to NK cell-mediate killing, the A375 cells and MUM2B cells were treated with NSC663284 and then co-cultured with NK92MI cells. 7-AAD staining assays demonstrated a significant increase in cell death in both A375 and MUM2B cells (Fig. 7H, I). Meanwhile, CCK-8 assays suggested that CDC25A inhibition enhance NK cell-mediated killing of melanoma cells (Fig. 7J). Together, these results indicate that CDC25A plays a role in the resistance of melanoma cells to NK cell-mediate killing. CDC25A inhibition may represent a promising strategy to enhance NK cell-mediated immunotherapy.

## Discussion

Despite significant advancements in genomic and proteomic technologies that have greatly enhanced our comprehension of tumors, effective biomarkers for treatment response and drug resistance are still lacking. CDC25 phosphatases, as critical regulators of the cell cycle, have been implicated in tumorigenesis since their initial discovery [26]. Further investigation into the correlation between CDC25 family members and cancer has revealed some members as potential prognostic indicators. In this study, we comprehensively evaluated the pivotal roles of CDC25 family in diagnosis, prognosis, and therapy, followed by an in-depth exploration of melanoma.

Initially, we analyzed the expression patterns of CDC25 family members at the transcription level. We observed elevated CDC25 family members expression in malignant cell microregions, macrophages, followed by endothelial cells and neutrophil. CDC25 family members were commonly overexpressed in 14 cancer types (CESC, CHOL, COAD, DLBC, ESCA, GBM, HNSC, LGG, LIHC, PAAD, PCCG, READ, SARC, SKCM, THYM), consistent with previous reports [7, 22, 27–30]. Notably, CDC25 family members, as tumor promoters, were lowly expressed in a limited number of cancers. The expression of CDC25A was downregulated in KIRC, while CDC25B expression was reduced in KICH, LUAD, LUSC, OV, PRAD, THCA and UCEC. Additionally, CDC25C expression exhibited downregulation in TGCT. The different expression patterns of CDC25 family members in different tumor types are speculated to be related to their molecular characteristics, epigenetic regulation, and tumor microenvironment. Our result showed that the methylation level of CDC25A promoter in kidney renal clear cell carcinoma was higher than in normal tissue. Promoter hypermethylation is associated with transcriptional quiescence in a variety of pathological and nonpathological contexts [31]. Therefore, we speculate that the downregulation of CDC25A expression in kidney renal clear cell carcinoma is regulated by methylation modification. The decreased expression level of CDC25B in lung squamous cell carcinoma and prostate adenocarcinoma may also be responsible for this. Mitochondrial dysfunction is one of the potential mechanisms of kidney chromophobe pathogenesis, which leads to continuous activation of AMPK, which in turn inhibits mTORC1 signaling and ultimately down-regulates CDC25B expression [32, 33]. However, study by Ngan et al. reported that CDC25B expression was frequently upregulated in human prostate adenocarcinoma tissue, and was positively correlated with high Gleason scores and advanced stages [34]. This discrepancy may arise from the fact that CDC25B overexpression can promote the AF2 domain of the AR to recruit CDC25B, thereby enhancing AR activation in prostate adenocarcinoma. This shift may result in the upregulation of hormone target gene expression, ultimately promoting abnormal cell proliferation. Thus, the development and progression of prostate adenocarcinoma are regulated by the complementary and/or synergistic effects of CDC25B overexpression [19, 34]. Wu et al. reported CDC25B overexpression in non-small cell lung cancer, however, this finding conflicts with our result [35]. Chen et al. found that high CDC25B expression had a p53-dependent tumor-suppressive effect in lung cancer. With the tumor progression, chromosome abnormalities accumulation might induce the inactivation of p53 and promote further tumor progression, which further increases the expression of CDC25B [36].

Previous studies have shown a significant association between high CDC25 expression and poor prognosis in most cancers, consistent with our findings. However, our study also indicated elevated CDC25 family members' expression correlates with prolonged survival in gastric carcinoma patients, which may suggest diverse roles of CDC25 family members in tumors. It was reported that the expression of PRIM1 was upregulated in gastric carcinoma, and it was correlated with poor prognosis. Silencing PRIM1 upregulated the expression of CDC25 and inhibited cell proliferation. Thus, the high expression level of CDC25 may associated with prolonged survival of gastric carcinoma patients [37]. These results reveal the complex effects of CDC25 family members on patients’ survival.

Two basic ingredients determine cell identity: genetic makeup and epigenetic modification [38]. Alterations of genetic makeup, including mutations, deletions, and amplifications, are commonly detected in tumor cells and are closely linked to biological functions, tumorigenesis, and development. Therefore, we analyzed the mutational profile of CDC25 family in human cancers based on TCGA datasets. The results showed ~4–20% of patients experienced CDC25 family member mutations in CESC, COAD, LUAD, SKCM, STAD, and UCEC, with missense mutations being the most common. High mutation frequency of CDC25 family members occurred in UCEC and SKCM. Together, the frequency of different mutational processes varies among cancer types. DNA methylation, a vital form of epigenetic modification, regulates gene expression and maintains genome stability. Previous research has suggested that aberrant methylation is a hallmark of cancer cells [38, 39]. Our study estimated the promoter DNA methylation levels of CDC25 family members and then explored the correlation between CDC25 family methylation and OS in diverse tumor types. The results revealed altered CDC25 methylation levels in three cancer types: KIRP, LUSC, and PRAD. Cox regression revealed high heterogeneity across different tumors, suggesting that CDC25 family members play various roles in tumor prognosis.

Based on our pan-cancer analysis, melanoma was selected as a representative cancer to confirm the expression and function of CDC25 family members. Our study first investigated the expression patterns and clinical associations of CDC25 family members using RNA-seq data. The results showed elevated CDC25 family members' expression in tumor tissues, particularly in Ki67 + + tumor and metastasis sites. High expression of CDC25A/C was a bad prognostic factor in melanoma. Spatial transcriptome analysis revealed a significant positive correlation between CDC25 family members' expression and tumor cell content. The methylation analysis of CDC25 family members indicated that the expression of CDC25A had a significantly negative correlation with its methylation level. Meanwhile, survival analysis revealed that the subgroup with high methylation and high expression exhibited significantly poorer prognosis compared to the other subgroups (log-rank test *p* < 0.05), indicating that the synergistic effect between CDC25A methylation and expression level status exerts an important influence on disease progression.

CDC25 family members play critical roles in key biological processes such as the cell cycle, DNA damage response, DNA repair, and cellular senescence. CDC25A regulated REGγ function by dephosphorylating NIP30, a mechanism that regulated p21 protein levels in a p53-independent manner following DNA damage [40]. In senescent HMECs, the reduction of CDC25A contributed to cyclin E/CDK2 inhibition and G1 arrest at senescent [16]. Zheng et al. found that upregulation of CDC25B expression can maintain peroxidation-antioxidant homeostasis in LECs, and prevent the cell damage caused by oxidative stress by activating the Akt/Nrf2 pathway and increasing various downstream antioxidant enzyme levels. It indicates that CDC25B activity may be affected by the downstream signaling pathway of metformin-related to the senescence mechanism [41, 42]. In addition, as key regulators of the cell cycle, CDC25 family members may be related to various cell death processes, such as apoptosis and entosis. Overexpression of CDC25 family members induces premature mitotic entry by circumventing checkpoint controls, thereby promoting chromosomal instability manifested as aneuploidy and/or mitotic errors, culminating in the production of genetically aberrant progeny. These aberrant progenies may engage cell competition pathways, thereby facilitating their elimination via entosis-mediated neighbor cell phagocytosis, a quality control mechanism that preserves genomic homeostasis [43]. And CDC25 family members may modulate cell adhesion and contractility through the regulation of Rho GTPases or cytoskeleton-associated proteins, consequently influencing entosis occurrence [12, 44, 45]. In addition to participating in various physiological processes, CDC25 family members also play an important role in the growth, metastasis, and invasion of various tumors [46–49]. Our study investigated the relationship between CDC25 family members' expression levels and cancer functional states in melanoma and revealed aan ssociation with cell cycle regulation, proliferation, and DNA damage response. These findings indicate their critical role in melanoma progression.

Further analysis showed that CDC25A, not CDC25B or CDC25C, plays a more dominant role in melanoma development. To confirm the function of CDC25A, we inhibited its activity using the NSC663284 inhibitor in two melanoma cell lines. Our results revealed that CDC25A inhibition suppressed cell proliferation and disrupted the cell cycle in melanoma cells.

Melanoma patients are typically treated with surgical excision in the early stages [50]. However, because melanoma onset is often insidious, many patients are diagnosed in advanced stages, where surgical resection is usually ineffective. Currently, a variety of drugs have been widely used in clinical melanoma therapy, including dacarbazine, temozolomide, and paclitaxel [51, 52]. Our results demonstrated that melanoma cell was more sensitive to doxorubicin when CDC25A was lowly expressed. We also confirmed that CDC25A inhibition could increase the sensitivity of melanoma to doxorubicin in vitro, suggesting that CDC25A may be a potential synthetic target for doxorubicin treatment in melanoma. With the continuous development of melanoma research, new therapeutic strategies, including immunotherapy, targeted therapy, and combination therapy, are emerging [7]. Additionally, our findings indicate that CDC25A contributes to melanoma cells’ resistance to NK cell-mediated killing. Inhibition of CDC25A may present an opportunity to improve NK cell-mediated immunotherapy.

In conclusion, our study unveils the comprehensive roles of CDC25 family members in cancer progression and clinical diagnosis, suggesting that CDC25 family members could serve as valuable prognostic biomarkers across various cancer types. Additionally, we confirmed that CDC25A inhibition enhanced the sensitivity of melanoma cells to chemotherapy and NK cell therapy. These findings may bring important implications for melanoma therapy. Meanwhile, there are some limitations in our work, with further studies required to explore the underlying mechanisms of CDC25A in regulating melanoma progression in vitro and in vivo.

## Materials and methods

### The related datasets

The high-throughput sequencing data and information involved in this paper are in Supplementary Table 1.

### Expression analysis

Bulk RNA-seq data: STAR-counts data and corresponding clinical information for 33 tumors were downloaded from the TCGA database (https://portal.gdc.cancer.gov). Data in TPM format were extracted, and normalization was performed using the log2(TPM + 1) transformation. RNA-seq data and corresponding clinical information were selected for further analysis. The GTEx data used in this study was from version V8; detailed information can be found on the official GTEx website (https://gtexportal.org/home/datasets).

The HPA (http://www.proteinatlas.org/), accessed on 25 February 2024, was used to examine CDC25A expression in normal and tumor tissues via immunohistochemistry staining.

Single-cell transcriptome data: Gene expression files at the single-cell resolution for pan-cancer were obtained from the TISCH database, and heatmaps of gene expression landscapes were constructed using the pheatmap package. Euclidean distance was used as a metric, and Ward’s method of minimum variance hierarchical clustering facilitated the identification of patterns and trends in the data, aiding in the recognition of conserved gene expression sources.

Spatial transcriptome data: Each microregion in the spatial transcriptome slice is defined by its predominant cell type. For example, when malignant cells comprise the highest proportion in a microregion, the microregion is designated as malignant; when endothelial cells dominate, the microregion is designated as endothelial. The average expression of genes in each cell type per slice was observed, *z*-score standardized using the scale function, and visualized using the pheatmap package.

### Survival analysis

A Cox regression analysis was conducted to assess the association between CDC25 family members' expression and OS, DSS, PFI, and DFI. We calculated the HR and its 95% confidence interval (CI) across different cancer types. Forest plots were used to analyze the association between CDC25 family members' expression and prognosis across various cancer types. The optimal cutoff values for the high-expression and low-expression groups were determined using the survminer package. To minimize potential biases due to over-grouping, we set a minimum ratio of high-expression to low-expression groups of no less than 0.3. The log-rank test was performed using the survfit function to evaluate the significance between the high-expression and low-expression groups.

ROC analysis was performed using the pROC package to calculate 95% CIs and the AUC to evaluate the diagnostic performance of gene expression between the tumor and normal groups. An AUC > 0.9 indicates high accuracy, 0.7–0.9 indicates moderate accuracy and 0.5–0.7 indicates low accuracy.

### DNA methylation analysis

The UCSCXenaShiny v2 database (DNMIVD) (https://shiny.hiplot.cn/ucsc-xena-shiny/) was used to analyze the DNA methylation status of CDC25 family members. Additionally, we examined the relationship between promoter methylation and CDC25 family members' expression across pan-cancer types and performed survival analysis to assess whether CDC25 family members affect methylation and prognosis. TSS200, TSS1500, 1stExone, and 5′UTR were included in the methylation analysis, and the median value was calculated to characterize the methylation level of each sample. Spearman correlation analysis investigates the relationship between methylation level and CDC25 family members' expression. The boxplot was used to visualize the methylation level at each site.

### Genetic alteration analysis

SNV data (*n* = 8663) were obtained from 33 cancer types in the TCGA database. The TCGA SNV dataset includes variant types such as Missense Mutation, Silent, 5′ Flank, 3′ UTR, RNA, In-Frame Deletion, Nonsense Mutation, Splice Site, Intron, 5′ UTR, In-Frame Insertion, Frame Shift Deletion, Nonstop Mutation, 3′ Flank, Frame Shift Insertion, and Translation Start Site. Silent, Intron, IGR, 3′ UTR, 5′ UTR, 3′ Flank, and 5′ Flank variants were excluded from the SNV percentage calculation. The SNV mutation frequency (percentage) for each gene’s coding region was calculated using the formula: Number of Mutated Samples/Number of Cancer Samples. SNV oncoplot visualizations were generated using the maftools package.

### Correlation analysis between CDC25 family members and tumor functional states

The CancerSEA database sorted out the different functional states of 14 tumor cells. The *z*-score algorithm was proposed by Lee et al. The activity of a given pathway was reflected by integrating the expression of characteristic genes, using the R package GSVA in the *z*-score parameter calculated the 14 functional state gene sets and obtained the combined *z*-score. We use the scale function to further standardize the score as the gene set score, and calculate the Pearson correlation between the CDC25 family members and each gene set score.

### Drug sensitivity analysis

The correlation between CDC25 family members' expression and drug sensitivity was evaluated using the Genomics of Drug Sensitivity in Cancer database (https://guolab.wchscu.cn/GSCA/#/drug). The half-maximal inhibitory concentration (IC50) values for melanoma patients in the TCGA dataset were calculated using the “pRRophetic” package.

### Cell culture

The liver cancer cell lines (Hep3B 2.17, Huh7, and PLC/PRF/5) and melanoma cell SK-MEL-28 were cultured in MEM (MACGENE Technology, Beijing, China) medium supplemented with 10% fetal bovine serum (Excell Bio) and 1% penicillin-streptomycin (MACGENE Technology, Beijing, China). MUM2B cells were maintained in RPMI 1640 (MACGENE Technology, Beijing, China) with 10% fetal bovine serum and 1% penicillin-streptomycin. NK92MI cell line was maintained in RPMI 1640 medium supplemented with 12.5% fetal bovine serum and 12.5% horse serum (Kangyuan Biology, China). The other cells (A375, A2058, MCF7, MDA-M-231, MDA-MB-436, and 293T) were cultured in DMEM (MACGENE Technology, Beijing, China) supplemented with 10% fetal bovine serum and 1% penicillin-streptomycin. All cells were incubated with 5% CO_2_ at 37 °C. All cells were obtained from ATCC and checked for mycoplasma using the GMyc-PCR Mycoplasma Test Kit (Yeasen Biotechnology).

### Antibodies and chemical reagents

Antibodies with working dilution factors, company source and catalog number include: anti-CDC25A (1:3000 for WB, 1:300 for IF, ProteinTech; #55031-1-AP), anti-CDC25B (1:3000 for WB, 1:300 for IF, ProteinTech; #28109-1-AP), anti-CDC25C (1:3000 for WB, 1:300 for IF, ProteinTech; #16485-1-AP), anti-beta-actin (1:6000, ProteinTech; #20536-1-AP), anti-CDK1/2 (1:3000, ABclonal; #A26696-PM), anti-p-CDK1/2 (1:1000, ABclonal; #AP1001), anti-E-cadherin (1:5000, ProteinTech; #20874-1-AP), anti-N-cadherin (1:3000, ProteinTech; #22018-1-AP), anti-Vimentin (1:50000, ProteinTech; #10366-1-AP), anti-cyclin A2 (1:10000, ProteinTech; #18202-1-AP), anti-cyclin B1 (1:2000, ProteinTech; #55004-1-AP), and anti-cyclin D1 (1:10000, ProteinTech; #26939-1-AP). Secondary antibodies include Alexa Fluor 488 anti-rabbit (1:500 for IF, Invitrogen; #A11008), anti-rabbit IgG HRP (1:5000, ProteinTech; #SA00001-2), and anti-mouse IgG HRP (1:5000, ProteinTech; # SA00001-1).

The CDC25A inhibitors NSC663284 (MCE, #HY-100034) and menadione (MCE, # HY-B0332) were dissolved in dimethyl sulfoxide at a concentration of 200 mM as a storage solution. Doxorubicin (MCE, #HY-15142) and paclitaxel (Solarbio, #SP8020) were dissolved in dimethyl sulfoxide, and the concentration of the storage solution was 100 mM.

### Plasmids and transfection

The short hairpin RNAs (shRNA) targeting CDC25A were cloned into the plko.1-U6-mcherry vector. The knockdown plasmids, along with the VSVG and D8.9 packaging vectors, were transfected into 293T cells using the jetPRIME transfection reagent (Polyplus-Transfection), according to the provided protocol. Lentivirus-infected tumor cells were selected by puromycin (2 μg/ml) for 48 h. Following selection, positive cells were identified via flow cytometry (Agilent Novocyte). The sequences for all shRNAs used in this study are provided in Supplementary Table 2.

### Immunofluorescence

Cells were fixed with 4% paraformaldehyde (PFA) and permeabilized with 0.2% Triton X-100 for 5 min, followed by blocking with 5% bovine serum albumin (BSA) for 1 h at room temperature. Primary antibodies, diluted in 5% BSA, were incubated with the samples overnight at 4 °C. After washing three times with 1× PBS for 10 min each, cells were incubated with secondary antibodies for 1 h at room temperature in the dark. Images were captured using the UltraView Vox confocal system (Perkin Elmer) on a Nikon Ti-E microscope.

### Western blot analysis

Cells were lysed with RIPA buffer containing a phosphatase inhibitor cocktail (1:100) and a protease inhibitor cocktail (1:100), and protein concentrations were quantified using a BCA protein assay kit (Thermo Fisher). Proteins were separated by SDS-PAGE, followed by transfer onto a 0.2 μm polyvinylidene fluoride membrane (Merck Millipore), and blocked with 5% non-fat milk for 1 h at room temperature. Primary antibodies were applied to the membrane overnight at 4 °C. The membranes were incubated with secondary antibodies at room temperature for 1 h after washing with 1× TBST buffer. The signal was detected using Immobilon chemiluminescence HRP substrate (Merck Millipore). ImageJ software was used to analyze the protein bands.

### Cell cycle

1 × 10^6^ cells were collected and fixed in ice-cold 70% ethanol at 4 °C overnight. Cells were washed with PBS and then centrifuged. The cells were then incubated with RNase A at 37 °C for 30 min, followed by staining with PI using a DNA content quantification assay (Solarbio, CA1510).

### Cell proliferation and cytotoxicity assays

5 × 10^3^ cells were seeded in 96-well plates and exposed to a gradient of drug concentrations. Cell viability was assessed using the CCK-8 kit (APEXBIO) according to the manufacturer’s instructions. The cytotoxicity of NK cells was assessed using 7-AAD staining. The 7-AAD cell viability assay (Beyotime, C1053S) was performed to carry out the experiment. The results were analyzed using flow cytometry (Agilent Novocyte).

### Statistical analysis

The data are presented as the mean ± standard deviation. Statistical analyses were conducted using GraphPad Prism 9 software. Statistical significance was assessed using Student’s *t*-test or two-way ANOVA. A *p* < 0.05 was considered statistically significant.

## Supplementary information

Supplemental Figure Legend

Supplemental Figure

Supplemental Table

Original western blots

## Data Availability

Links to the datasets used in this study are given in Supplementary Table 1. Results in validation experiments were all shown in section 2 parts 5–7.
