# Supplementary material for: CDC25A inhibition sensitizes melanoma cells to doxorubicin and NK cell therapy
Source: Cell Death Dis. 2025 Apr 11;16(1):276. doi: 10.1038/s41419-025-07598-w (PMC11992059; doi:10.1038/s41419-025-07598-w)
Supplement: Supplementary file 1 — Supplemental Figure Legend [file 41419_2025_7598_MOESM1_ESM.docx]

**Supplementary figure legend:**

**Figure S1 Single-cell RNA-seq analysis of CDC25 family.**

The expression landscape of CDC25 family genes across pan-cancer, at single-cell resolution, was retrieved from the TISCH database.

**Figure S2 Identifying CDC25 family members prognostic values.**

A. Immunohistochemical staining results of CDC25 family members in normal and corresponding cancer tissues, as provided by the HPA database.

B. ROC assesses the diagnostic efficacy of gene expression in the tumor.

**Figure S3 Methylation analysis in Pan-Cancer.**

A. The methylation landscape of CDC25 family genes across various cancers, comparing tumor and normal tissues.

**Figure S4 The clinical association analysis of CDC25 in SKCM tumor tissue.**

A. Univariate and multivariate regression analysis of CDC25 expression in the TCGA-SKCM cohort.
B. Differential expression of CDC25 between stages.
C, D, E. Cox proportional hazards regression of CDC25 expression and melanoma prognosis in the TCGA-SKCM and GSE65904 datasets (DSS & PFS).
F, G. High-resolution images of melanoma are organized.
H. Wilcoxon rank-sum tests were used to evaluate the statistical significance of gene expression differences between the three groups.
I. Spearman correlation analysis was performed to calculate the correlation between cell counts and gene expression in all spots.

**Figure S5 The correlation between CDC25 family members expression and hallmarks of cancers in SKCM.**

A-C. The correlation between CDC25 family members expression and 14 hallmarks of cancer (angiogenesis, apoptosis, cell cycle, differentiation, DNA damage, DNA repair, EMT, hypoxia, inflammation, invasion, metastasis, proliferation, quiescence, and stemness).

D. The relationship between CDC25A and cell cycle-related genes (CCNA2, CCNB1, CCND1) expression in SKCM.

E. The relationship between CDC25A and EMT-related genes (CDH1, CDH2, VIM) expression in SKCM.
