# Supplementary material for: CDC25A inhibition sensitizes melanoma cells to doxorubicin and NK cell therapy
Source: Cell Death Dis. 2025 Apr 11;16(1):276. doi: 10.1038/s41419-025-07598-w (PMC11992059; doi:10.1038/s41419-025-07598-w)
Supplement: Supplementary file 2 — Supplemental Figure [file 41419_2025_7598_MOESM2_ESM.pdf]

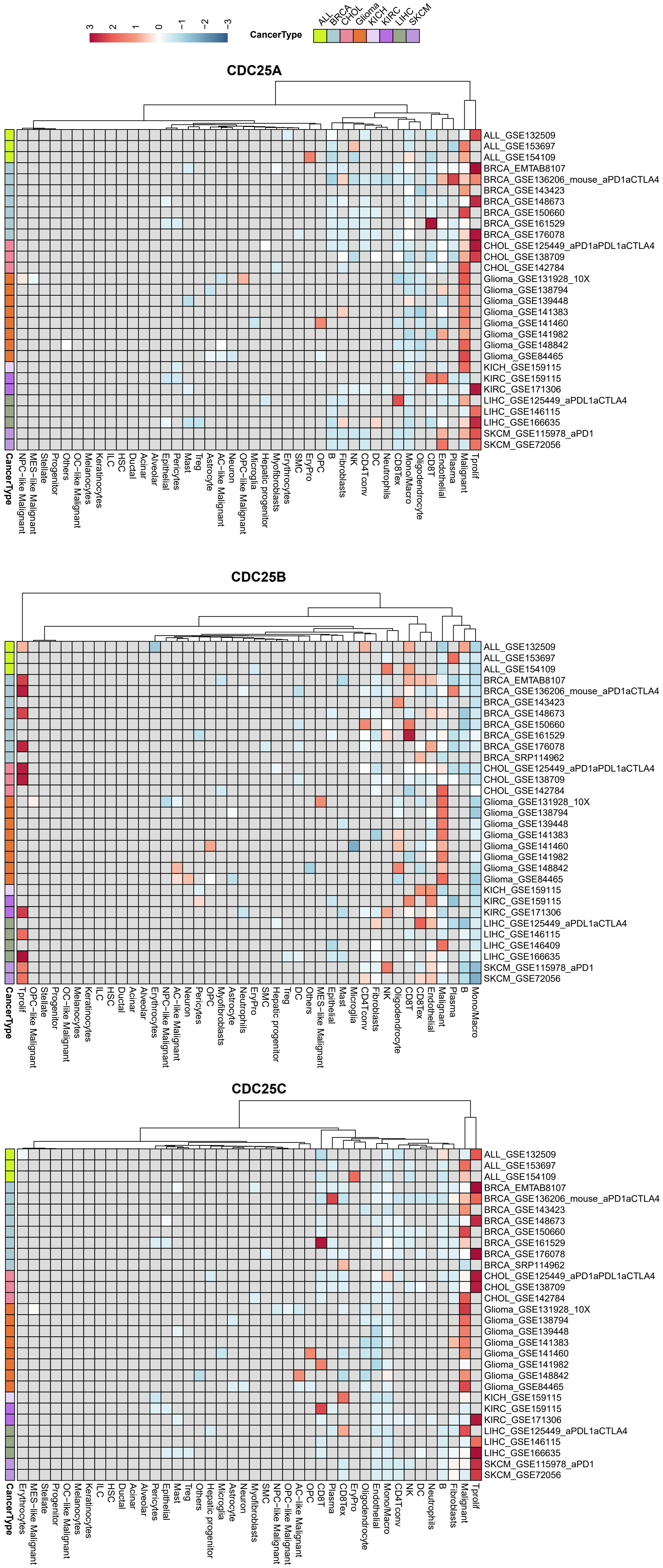

Figure S1 Single-cell RNA-seq analysis of CDC25 family.

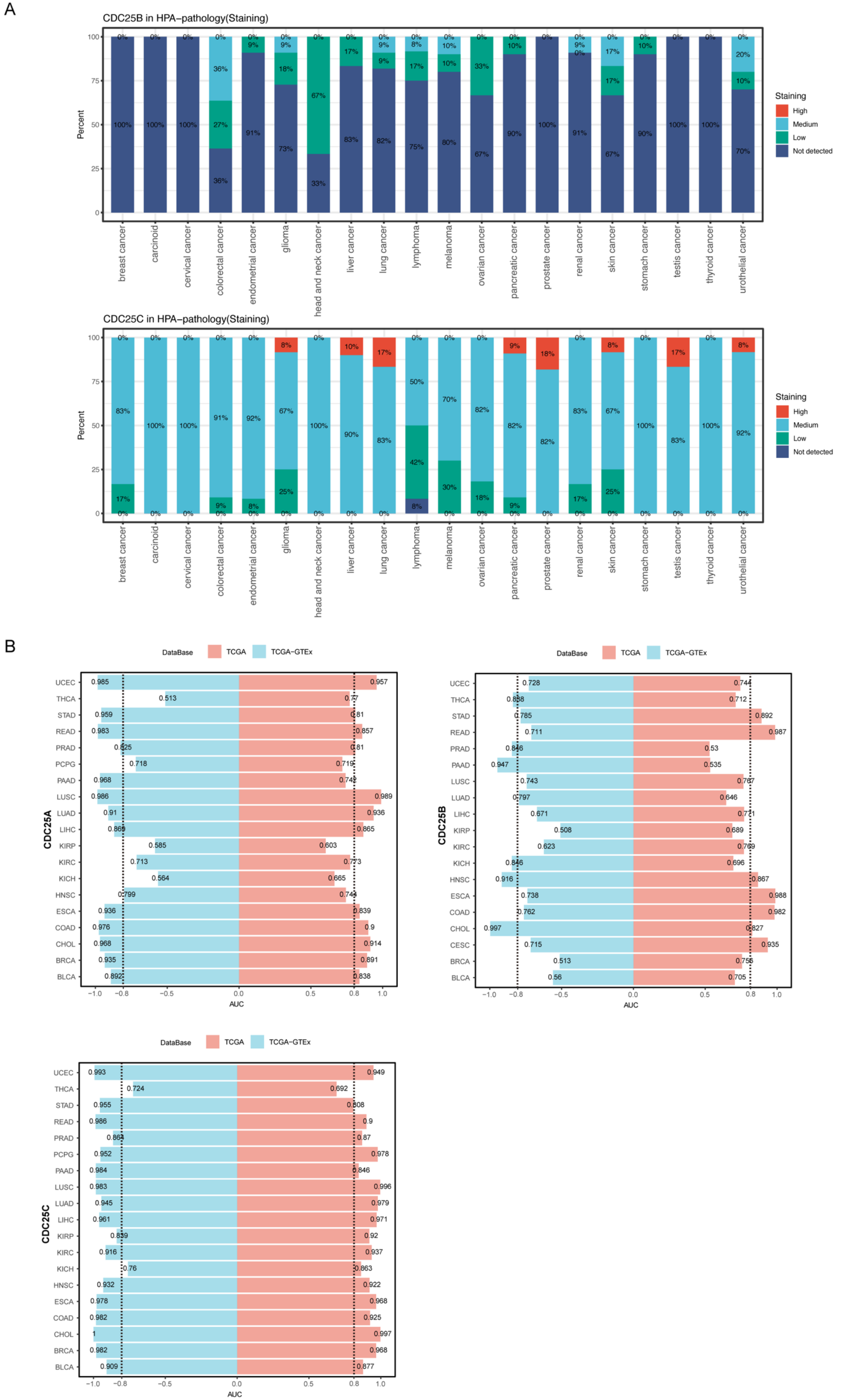

Figure S2 Identifying CDC25 family members prognostic values.

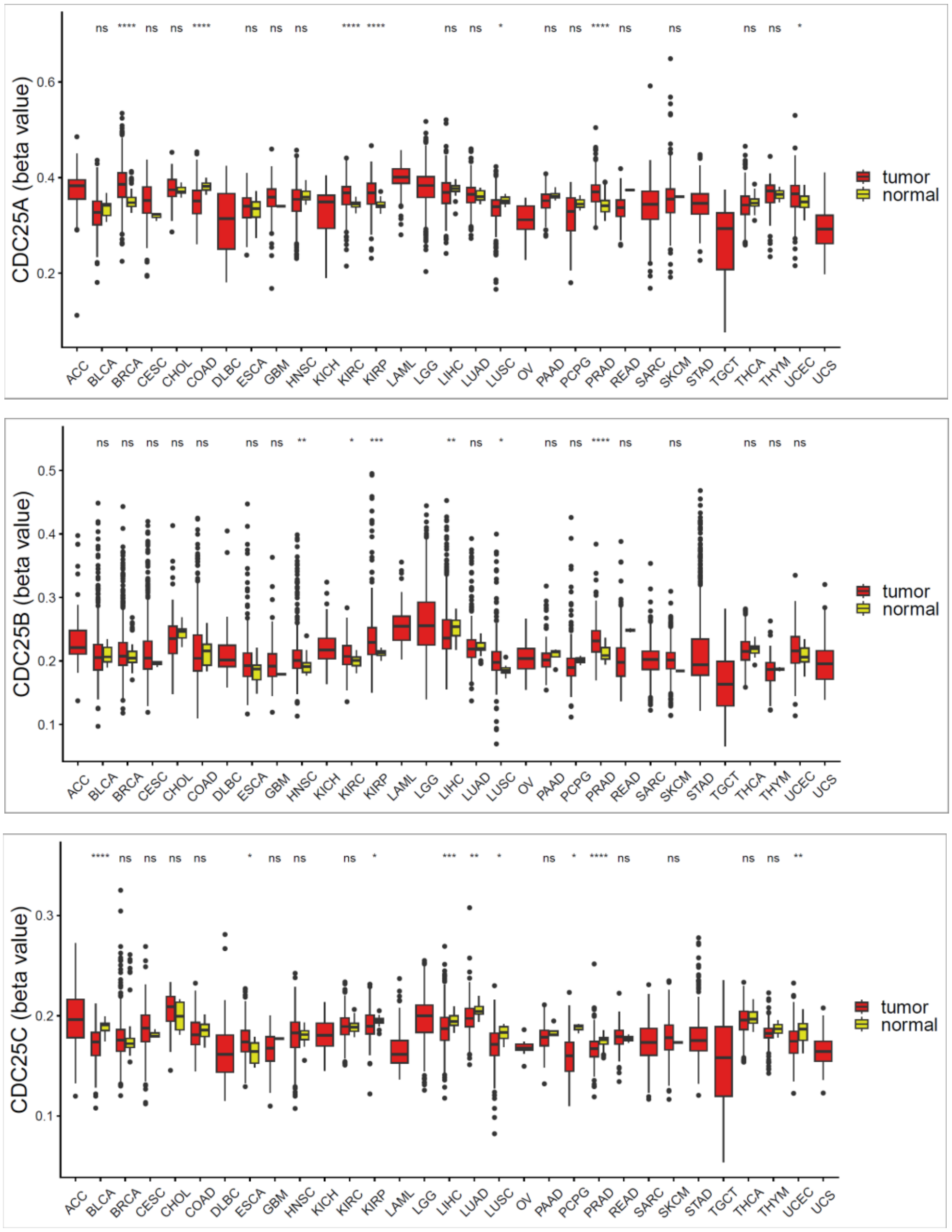

Figure S3 Methylation analysis in pan-cancer.

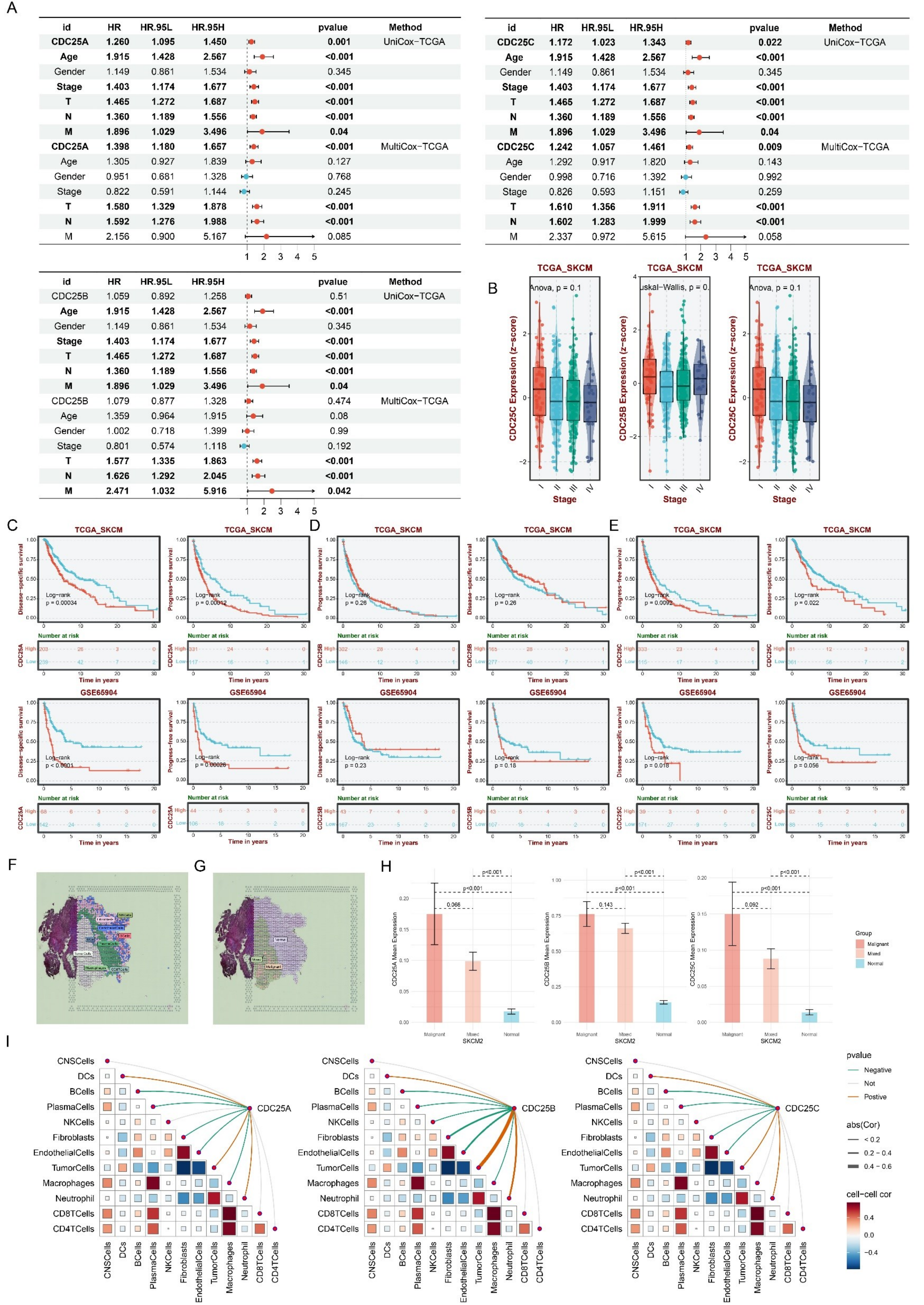

Figure S4 The Clinical association analysis of CDC25 in SKCM tumor tissue.

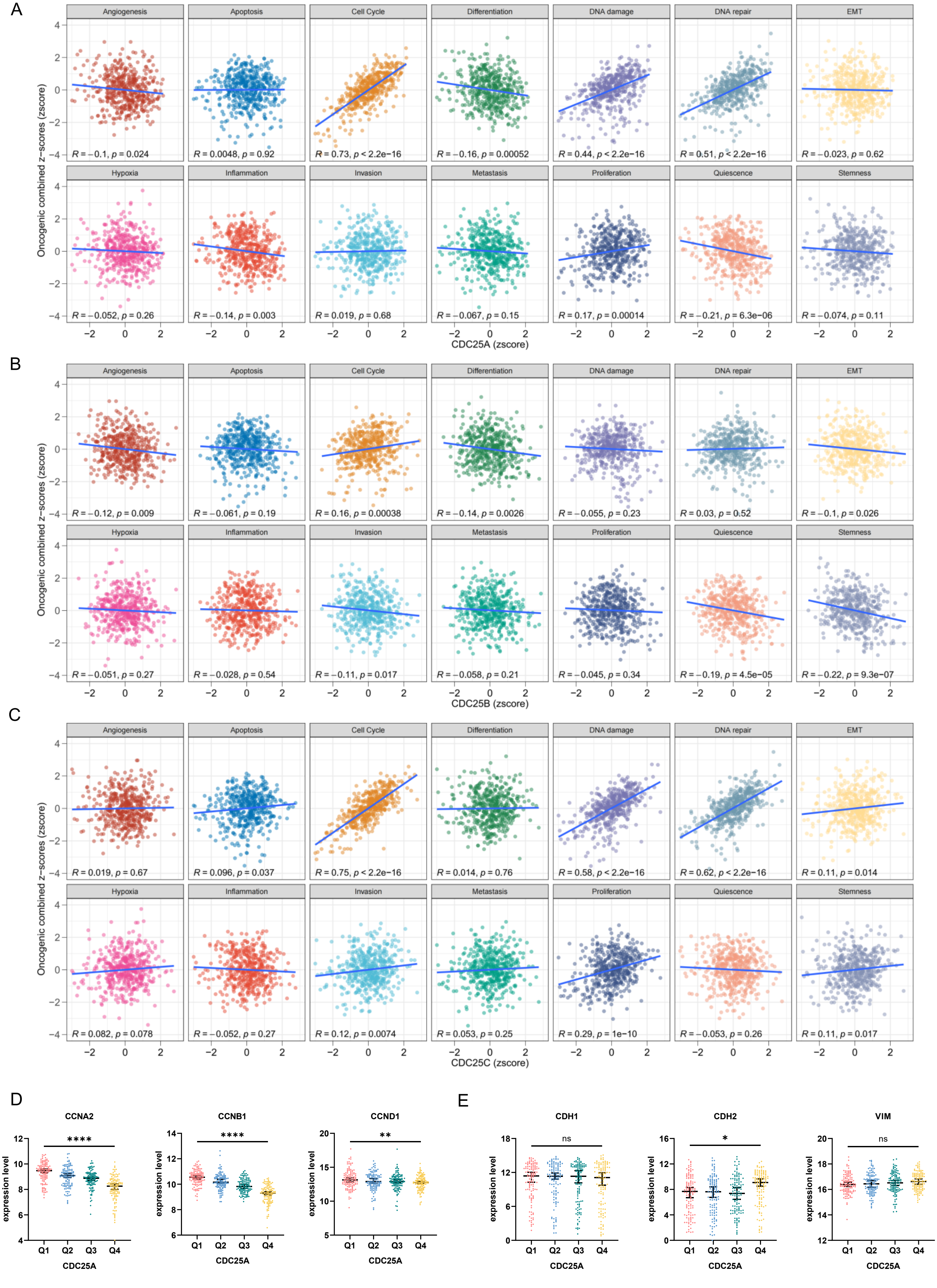

Figure S5 The correlation between CDC25 family members expression and hallmarks of cancer in SKCM.
