# Supplementary material for: CDC25A inhibition sensitizes melanoma cells to doxorubicin and NK cell therapy
Source: Cell Death Dis. 2025 Apr 11;16(1):276. doi: 10.1038/s41419-025-07598-w (PMC11992059; doi:10.1038/s41419-025-07598-w)
Supplement: Supplementary file 3 — Supplemental Table [file 41419_2025_7598_MOESM3_ESM.docx]

**Supplementary Table 1 List of the related datasets.**

| **Category** | **Datasets** | **Cancer type** | **Links** |
| --- | --- | --- | --- |
| Bulk RNA-seq/SNV data/Clinical data | TCGA-ACC | Adrenocortical carcinoma | https://www.cancer.gov/ccg/research/genome-sequencing/tcga |
|  | TCGA-BLCA | Bladder Urothelial Carcinoma |  |
|  | TCGA-BRCA | Breast invasive carcinoma |  |
|  | TCGA-CESC | Cervical squamous cell carcinoma and endocervical adenocarcinoma |  |
|  | TCGA-CHOL | Cholangiocarcinoma |  |
|  | TCGA-COAD | Colon adenocarcinoma |  |
|  | TCGA-DLBC | Lymphoid Neoplasm Diffuse Large B-cell Lymphoma |  |
|  | TCGA-ESCA | Esophageal carcinoma |  |
|  | TCGA-GBM | Glioblastoma multiforme |  |
|  | TCGA-HNSC | Head and Neck squamous cell carcinoma |  |
|  | TCGA-KICH | Kidney Chromophobe |  |
|  | TCGA-KIRC | Kidney renal clear cell carcinoma |  |
|  | TCGA-KIRP | Kidney renal papillary cell carcinoma |  |
|  | TCGA-LGG | Brain Lower Grade Glioma |  |
|  | TCGA-LIHC | Liver hepatocellular carcinoma |  |
|  | TCGA-LUAD | Lung adenocarcinoma |  |
|  | TCGA-LUSC | Lung squamous cell carcinoma |  |
|  | TCGA-OV | Ovarian serous cystadenocarcinoma |  |
|  | TCGA-PAAD | Lung squamous cell carcinoma |  |
|  | TCGA-PCPG | Pheochromocytoma and Paraganglioma |  |
|  | TCGA-PRAD | Prostate adenocarcinoma |  |
|  | TCGA-READ | Rectum adenocarcinoma |  |
|  | TCGA-SARC | Sarcoma |  |
|  | TCGA-SKCM | Skin Cutaneous Melanoma |  |
|  | TCGA-STAD | Stomach adenocarcinoma |  |
|  | TCGA-TGCT | Testicular Germ Cell Tumors |  |
|  | TCGA-THCA | Testicular Germ Cell Tumors |  |
|  | TCGA-THYM | Thymoma |  |
|  | TCGA-UCEC | Uterine Corpus Endometrial Carcinoma |  |
|  | TCGA-UCS | Uterine Carcinosarcoma |  |
| Bulk RNA-seq/Clinical data | GSE46517 | Skin Cutaneous Melanoma | https://www.ncbi.nlm.nih.gov/geo/query/acc.cgi?acc= GSE46517 |
|  | GSE98394 |  | https://www.ncbi.nlm.nih.gov/geo/query/acc.cgi?acc= GSE98394 |
|  | GSE22153 |  | https://www.ncbi.nlm.nih.gov/geo/query/acc.cgi?acc= GSE22153 |
|  | GSE100797 |  | https://www.ncbi.nlm.nih.gov/geo/query/acc.cgi?acc= GSE100797 |
|  | GSE133713 |  | https://www.ncbi.nlm.nih.gov/geo/query/acc.cgi?acc= GSE133713 |
|  | GSE22153 |  | https://www.ncbi.nlm.nih.gov/geo/query/acc.cgi?acc= GSE22153 |
|  | GSE65904 |  | https://www.ncbi.nlm.nih.gov/geo/query/acc.cgi?acc= GSE65904 |
|  | GSE98394 |  | https://www.ncbi.nlm.nih.gov/geo/query/acc.cgi?acc= GSE98394 |
| Spatial transcriptome | CRC1 | Colorectal cancer | https://www.10xgenomics.com/cn/datasets/human-colorectal-cancer-whole-transcriptome-analysis-1-standard-1-2-0 |
|  | CRC10 |  | https://aacrjournals.org/cancerdiscovery/article/12/1/134/675646/Spatiotemporal-Immune-Landscape-of-Colorectal |
|  | CRC2 |  | https://www.10xgenomics.com/cn/datasets/human-intestine-cancer-1-standard |
|  | CRC3 |  | https://aacrjournals.org/cancerdiscovery/article/12/1/134/675646/Spatiotemporal-Immune-Landscape-of-Colorectal |
|  | CRC4 |  | https://aacrjournals.org/cancerdiscovery/article/12/1/134/675646/Spatiotemporal-Immune-Landscape-of-Colorectal |
|  | CRC5 |  | https://aacrjournals.org/cancerdiscovery/article/12/1/134/675646/Spatiotemporal-Immune-Landscape-of-Colorectal |
|  | CRC6 |  | https://aacrjournals.org/cancerdiscovery/article/12/1/134/675646/Spatiotemporal-Immune-Landscape-of-Colorectal |
|  | CRC7 |  | https://aacrjournals.org/cancerdiscovery/article/12/1/134/675646/Spatiotemporal-Immune-Landscape-of-Colorectal |
|  | CRC8 |  | https://aacrjournals.org/cancerdiscovery/article/12/1/134/675646/Spatiotemporal-Immune-Landscape-of-Colorectal |
|  | CRC9 |  | https://aacrjournals.org/cancerdiscovery/article/12/1/134/675646/Spatiotemporal-Immune-Landscape-of-Colorectal |
|  | LIHC1 | Liver cancer | https://www.ncbi.nlm.nih.gov/geo/query/acc.cgi?acc=GSM6177612 |
|  | LIHC2 |  | https://linkinghub.elsevier.com/retrieve/pii/S0168827823000235 |
|  | LIHC3 |  | https://linkinghub.elsevier.com/retrieve/pii/S0168827823000235 |
|  | LIHC4 |  | https://linkinghub.elsevier.com/retrieve/pii/S0168827823000235 |
|  | LIHC5 |  | https://linkinghub.elsevier.com/retrieve/pii/S0168827823000235 |
|  | LIHC6 |  | https://linkinghub.elsevier.com/retrieve/pii/S0168827823000235 |
|  | LIHC7 |  | https://linkinghub.elsevier.com/retrieve/pii/S0168827823000235 |
|  | LIHC8 |  | https://linkinghub.elsevier.com/retrieve/pii/S0168827823000235 |
|  | LUAD1 | Lung adenocarcinoma | https://www.ncbi.nlm.nih.gov/geo/query/acc.cgi?acc=GSM5420754 |
|  | LUAD2 |  | https://www.ncbi.nlm.nih.gov/geo/query/acc.cgi?acc=GSM5420751 |
|  | LUAD3 |  | https://www.ncbi.nlm.nih.gov/geo/query/acc.cgi?acc=GSM5420749 |
|  | SKCM1 | Skin Cutaneous Melanoma | https://www.10xgenomics.com/cn/datasets/human-melanoma-if-stained-ffpe-2-standard |
|  | SKCM2 |  | https://www.ncbi.nlm.nih.gov/geo/query/acc.cgi?acc=GSM5420750 |
|  | UCEC | Uterine Corpus Endometrial Carcinoma | https://www.ncbi.nlm.nih.gov/geo/query/acc.cgi?acc=GSM6177623 |
| Single-cell transcriptome | ALL_GSE132509 | Acute lymphocytic leukemia | https://www.ncbi.nlm.nih.gov/geo/query/acc.cgi?acc=GSE132509 |
|  | ALL_GSE153697 |  | https://www.ncbi.nlm.nih.gov/geo/query/acc.cgi?acc= GSE153697 |
|  | ALL_GSE154109 |  | https://www.ncbi.nlm.nih.gov/geo/query/acc.cgi?acc= GSE154109 |
|  | BRCA_EMTAB8107 | Breast invasive carcinoma | https://www.ebi.ac.uk/biostudies/studies/E-MTAB-8107 |
|  | BRCA_GSE136206_mouse_aPD1aCTLA4 |  | https://www.ncbi.nlm.nih.gov/geo/query/acc.cgi?acc= GSE136206 |
|  | BRCA_GSE143423 |  | https://www.ncbi.nlm.nih.gov/geo/query/acc.cgi?acc= GSE143423 |
|  | BRCA_GSE148673 |  | https://www.ncbi.nlm.nih.gov/geo/query/acc.cgi?acc= GSE148673 |
|  | BRCA_GSE150660 |  | https://www.ncbi.nlm.nih.gov/geo/query/acc.cgi?acc= GSE150660 |
|  | BRCA_GSE161529 |  | https://www.ncbi.nlm.nih.gov/geo/query/acc.cgi?acc= GSE161529 |
|  | BRCA_GSE176078 |  | https://www.ncbi.nlm.nih.gov/geo/query/acc.cgi?acc= GSE176078 |
|  | BRCA_SRP114962 |  | https://trace.ncbi.nlm.nih.gov/Traces/?view=study&acc=SRP114962 |
|  | CHOL_GSE125449_aPD1aPDL1aCTLA4 | Cholangiocarcinoma | https://www.ncbi.nlm.nih.gov/geo/query/acc.cgi?acc= GSE125449 |
|  | CHOL_GSE138709 |  | https://www.ncbi.nlm.nih.gov/geo/query/acc.cgi?acc= GSE138709 |
|  | CHOL_GSE142784 |  | https://www.ncbi.nlm.nih.gov/geo/query/acc.cgi?acc= GSE142784 |
|  | Glioma_GSE131928_10X | Glioma | https://www.ncbi.nlm.nih.gov/geo/query/acc.cgi?acc= GSE131928 |
|  | Glioma_GSE138794 |  | https://www.ncbi.nlm.nih.gov/geo/query/acc.cgi?acc= GSE138794 |
|  | Glioma_GSE139448 |  | https://www.ncbi.nlm.nih.gov/geo/query/acc.cgi?acc= GSE139448 |
|  | Glioma_GSE141383 |  | https://www.ncbi.nlm.nih.gov/geo/query/acc.cgi?acc= GSE141383 |
|  | Glioma_GSE141460 |  | https://www.ncbi.nlm.nih.gov/geo/query/acc.cgi?acc= GSE141460 |
|  | Glioma_GSE141982 |  | https://www.ncbi.nlm.nih.gov/geo/query/acc.cgi?acc= GSE141982 |
|  | Glioma_GSE84465 |  | https://www.ncbi.nlm.nih.gov/geo/query/acc.cgi?acc= GSE84465 |
|  | KICH_GSE159115 | Kidney Chromophobe | https://www.ncbi.nlm.nih.gov/geo/query/acc.cgi?acc= GSE159115 |
|  | KIRC_GSE159115 | Kidney renal clear cell carcinoma | https://www.ncbi.nlm.nih.gov/geo/query/acc.cgi?acc= GSE159115 |
|  | KIRC_GSE171306 |  | https://www.ncbi.nlm.nih.gov/geo/query/acc.cgi?acc= GSE171306 |
|  | LIHC_GSE125449_aPDL1aCTLA4 | Liver hepatocellular carcinoma | https://www.ncbi.nlm.nih.gov/geo/query/acc.cgi?acc= GSE125449 |
|  | LIHC_GSE146115 |  | https://www.ncbi.nlm.nih.gov/geo/query/acc.cgi?acc= GSE146115 |
|  | LIHC_GSE146409 |  | https://www.ncbi.nlm.nih.gov/geo/query/acc.cgi?acc= GSE146409 |
|  | LIHC_GSE166635 |  | https://www.ncbi.nlm.nih.gov/geo/query/acc.cgi?acc= GSE166635 |
|  | SKCM_GSE115978_aPD1 | Skin Cutaneous Melanoma | https://www.ncbi.nlm.nih.gov/geo/query/acc.cgi?acc= GSE115978 |
|  | SKCM_GSE72056 |  | https://www.ncbi.nlm.nih.gov/geo/query/acc.cgi?acc= GSE72056 |

**Supplementary Table 2 List of shRNAs.**

| **Name** | **Sequence (5’-3’)** |
| --- | --- |
| shCDC25A-1 | CCGGGCCCGTCGTGAAGGCGCTACTCGAGTAGCGCCTTCACGACGGGCTTTTT |
| shCDC25A-4 | CCGGGCTGTTGGGATGTAGTCCACTCGAGTGGACTACATCCCAACAGCTTTTT |
| shCDC25A-7 | CCGGGCAGTGAACCAGGGAATTTCACTCGAGTGAAATTCCCTGGTTCACTGCTTTTT |
| shCDC25A-8 | CCGGGCTGGGAAACATCAGGATTTACTCGAGTAAATCCTGATGTTTCCCAGCTTTTT |
| shNC | CACCGTTCTCCGAACGTGTCACGT TTCAAGAGA ACGTGACACGTTCGGAGAATTTTTT |
