# Supplementary figures and images for: CDC25A inhibition sensitizes melanoma cells to doxorubicin and NK cell therapy
Source: Cell Death Dis. 2025 Apr 11;16(1):276. doi: 10.1038/s41419-025-07598-w (PMC11992059; doi:10.1038/s41419-025-07598-w)

Fig.1D

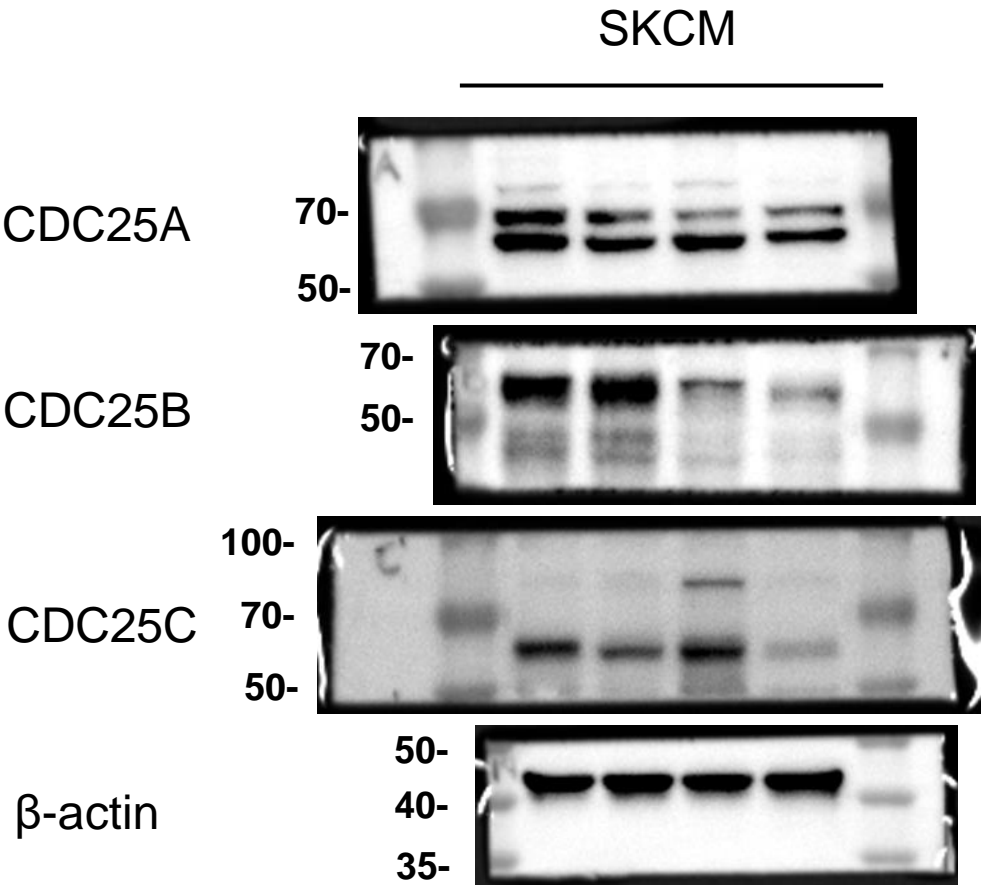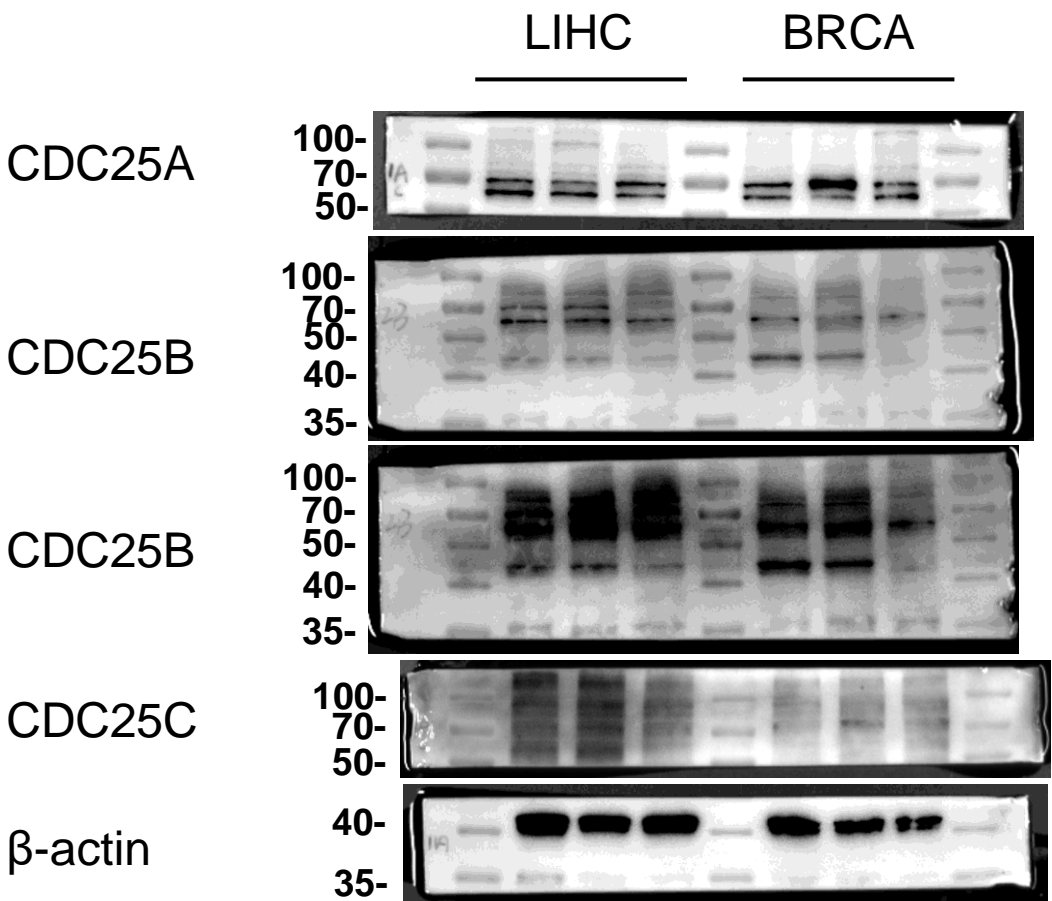

Fig.4C

MUM2B

shNC shCDC25A-1 shCDC25A-4 shCDC25A-7 shCDC25A-8

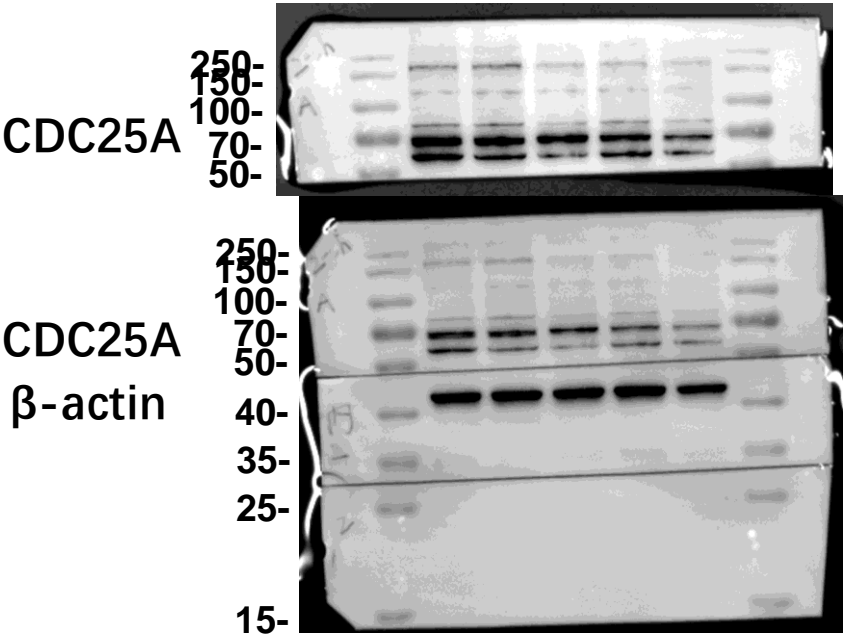

Fig.5C

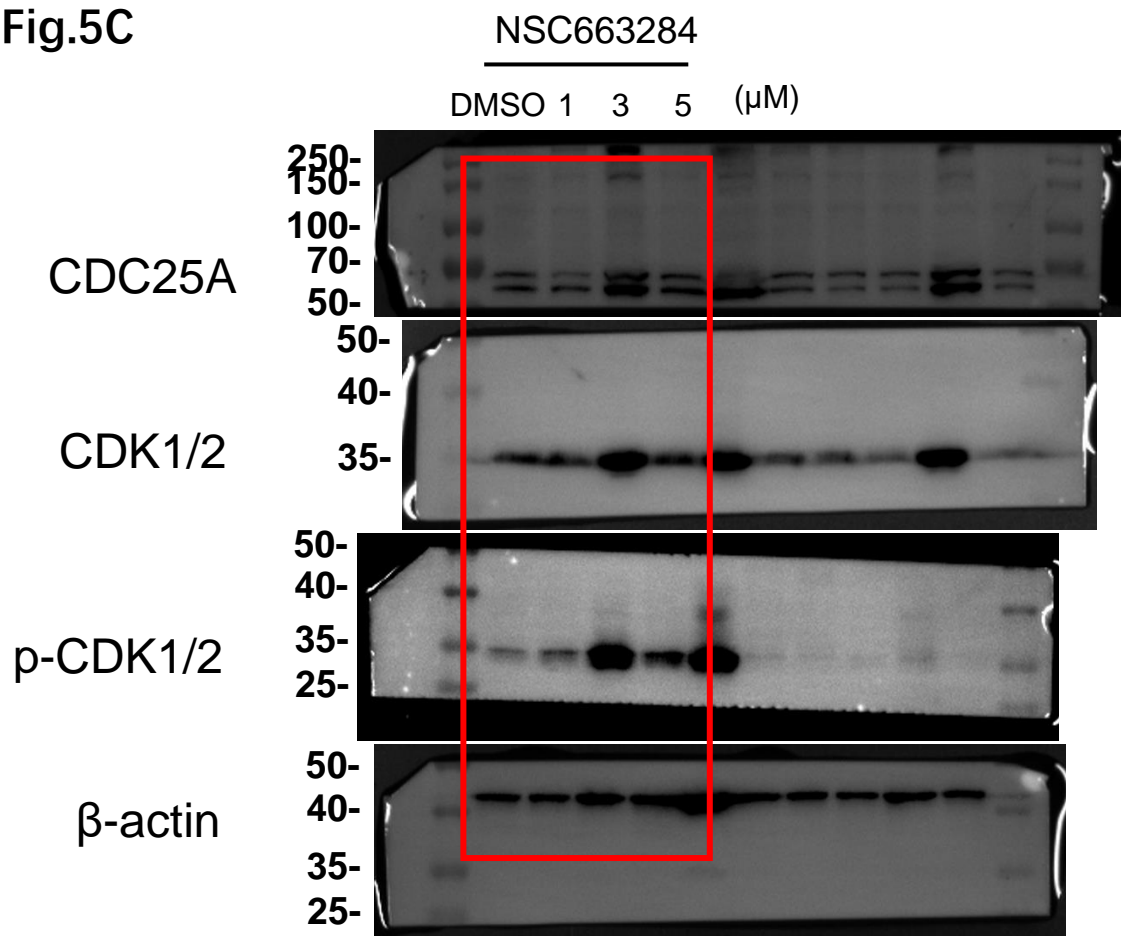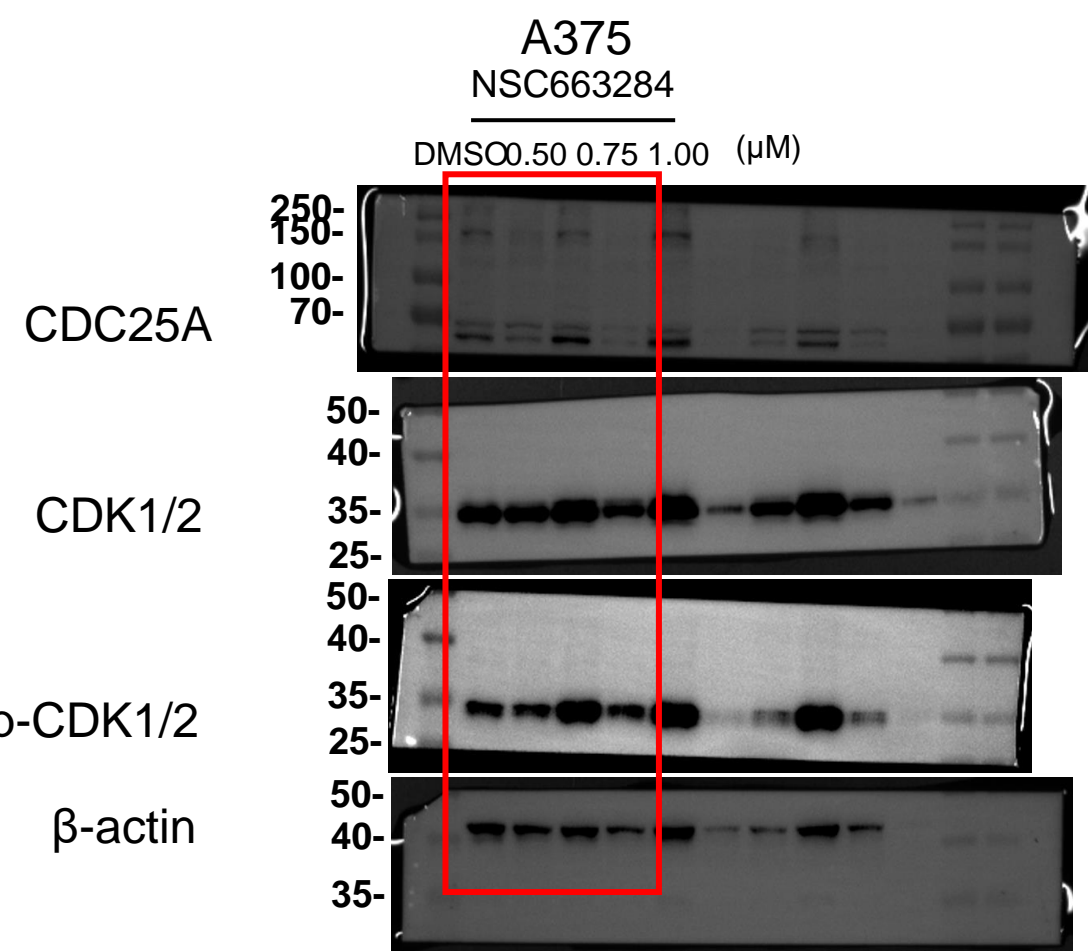

MUM2B

Fig.5G

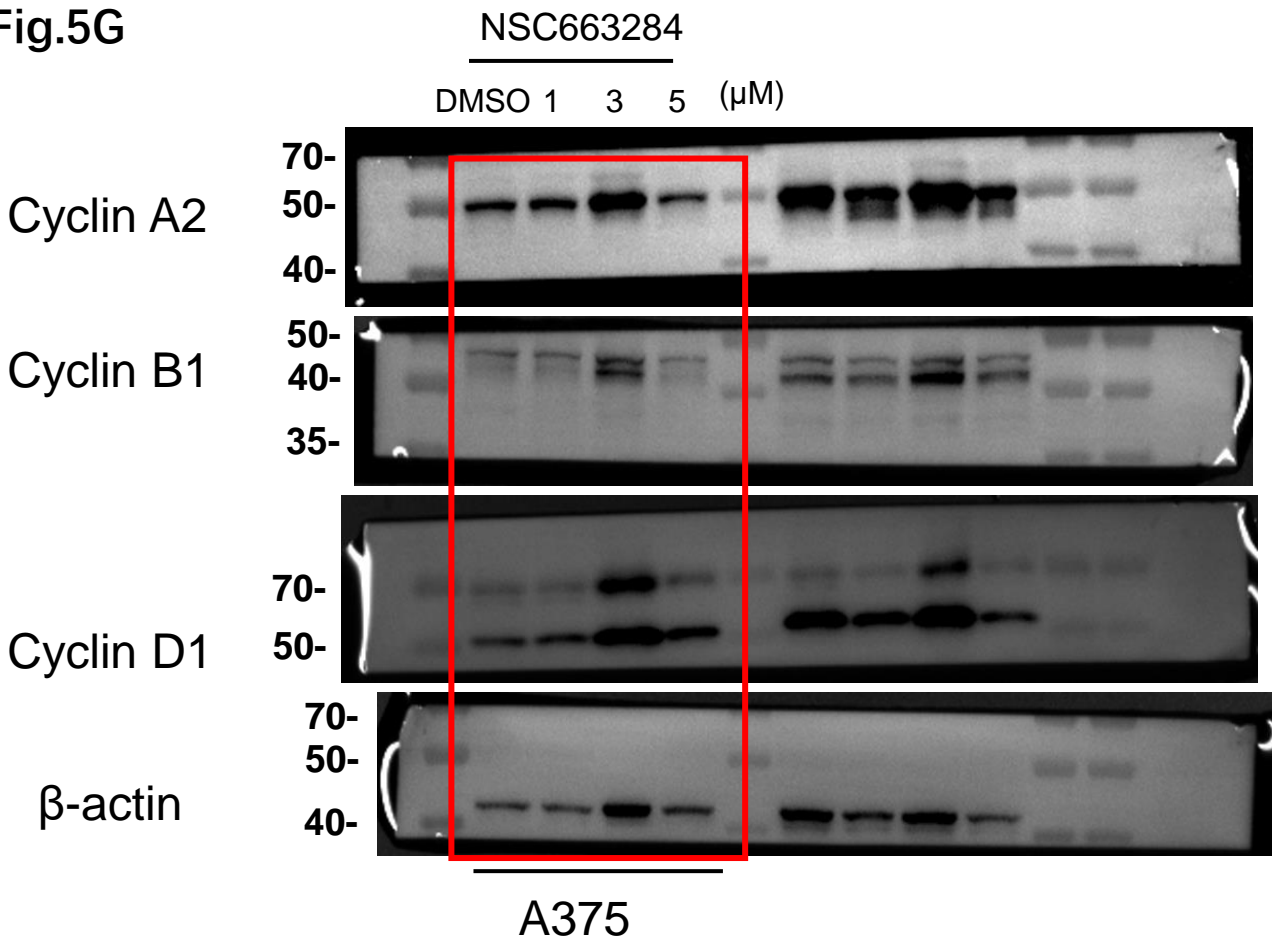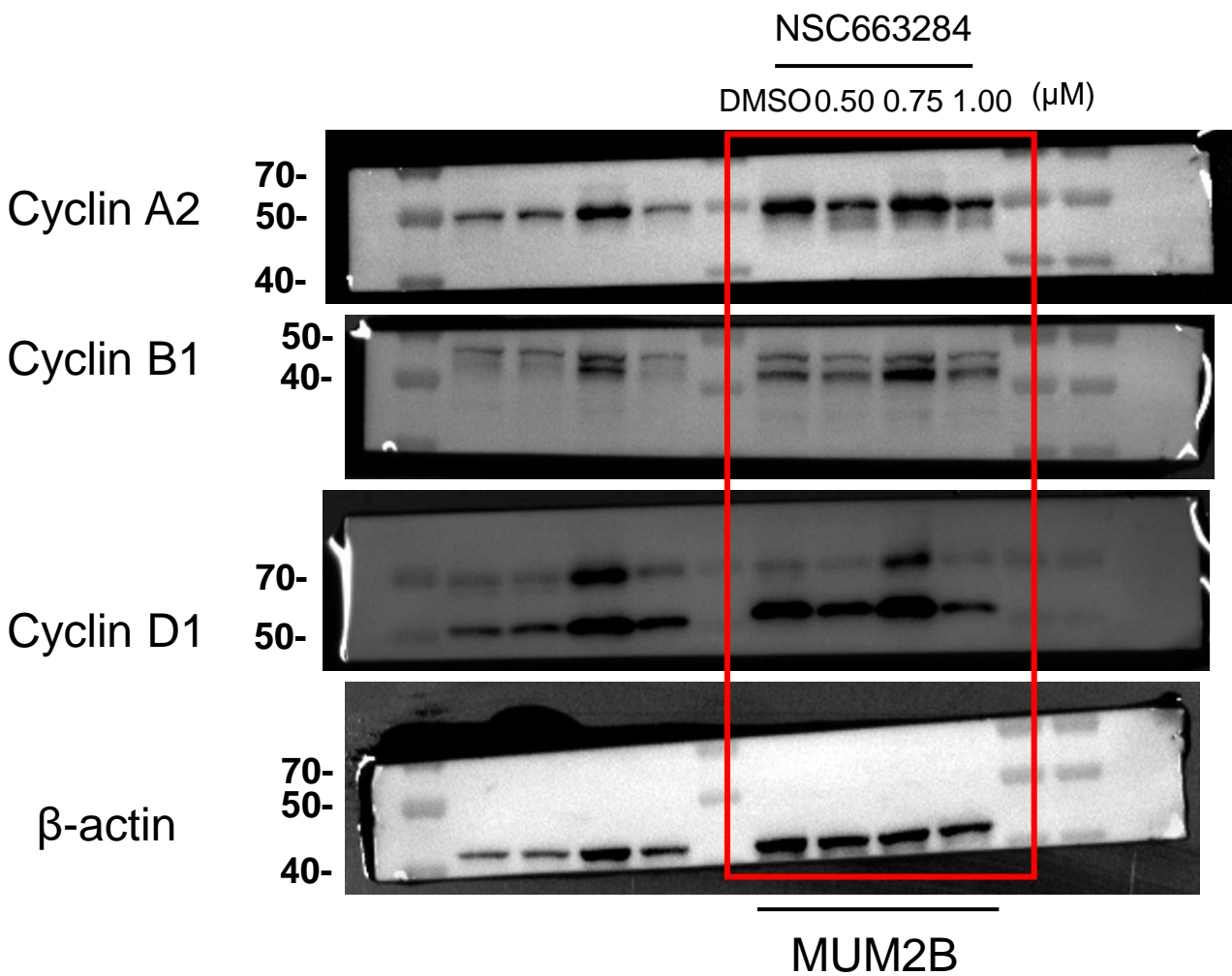

Fig.5H

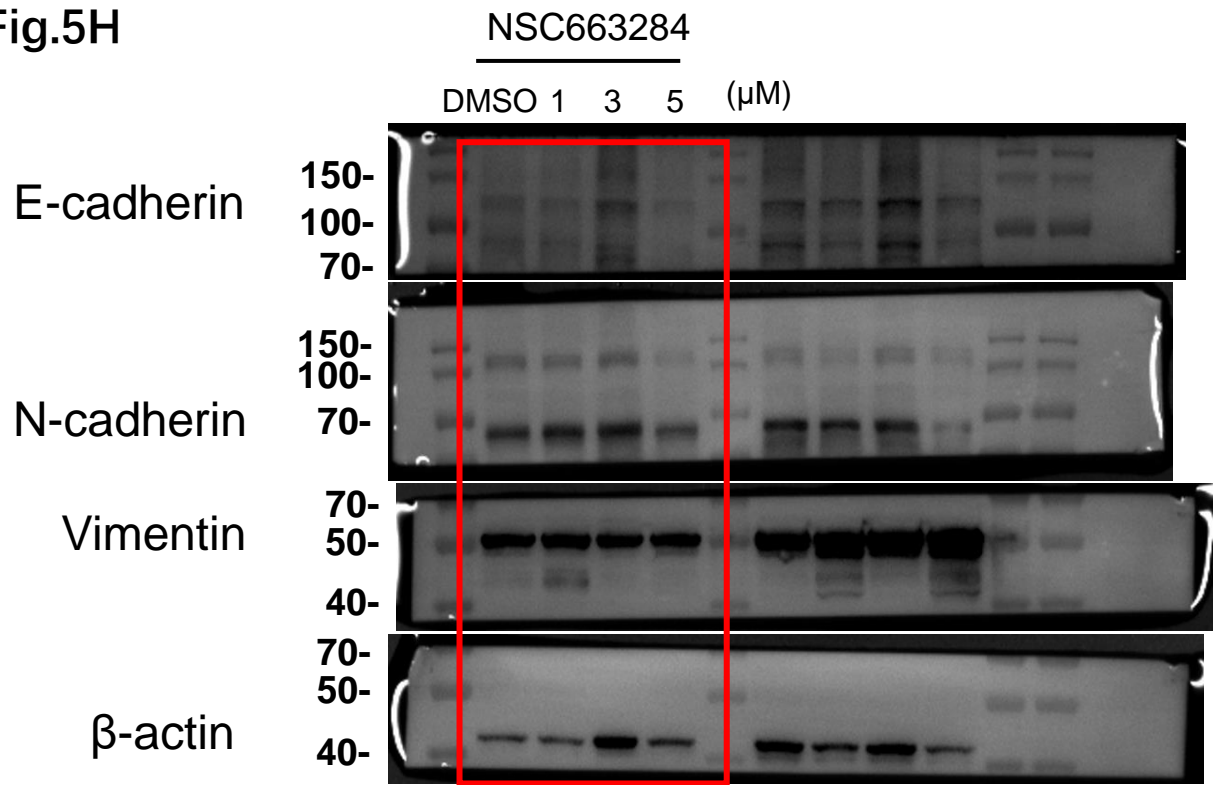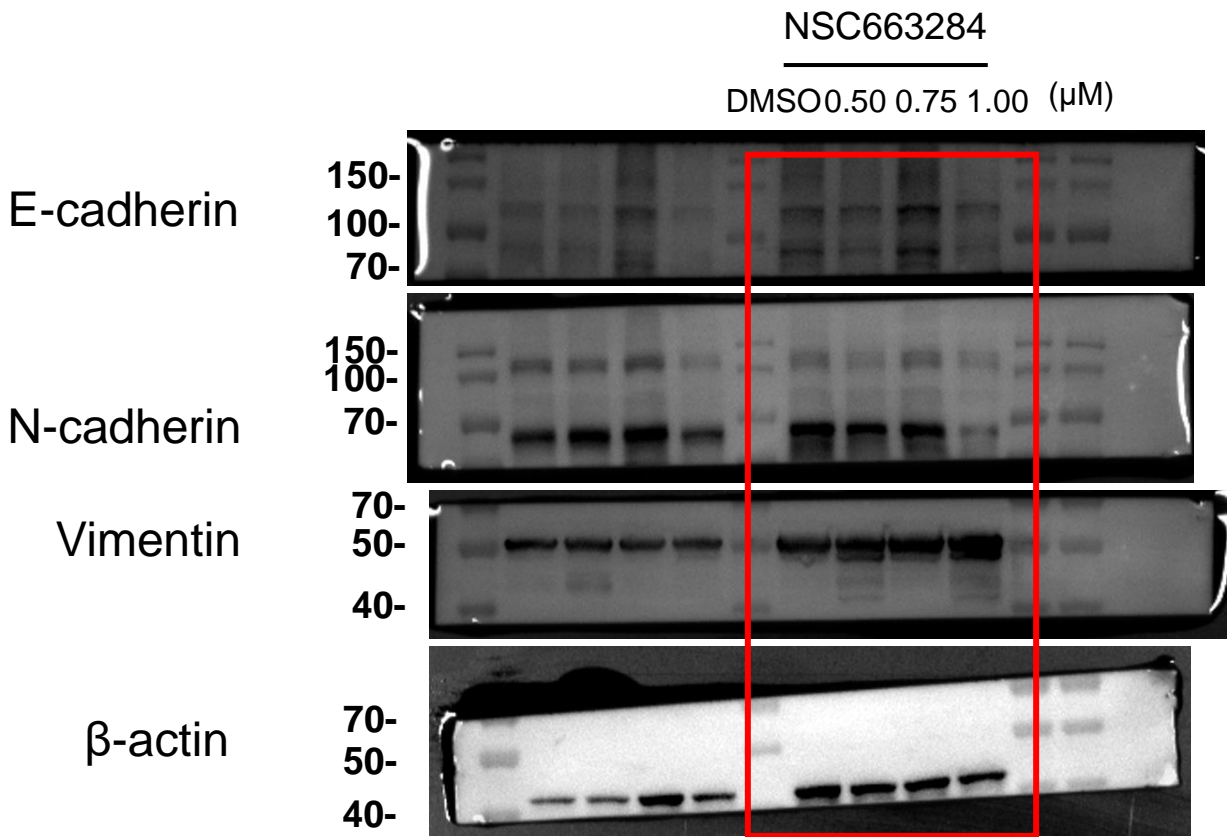

Supplement: Supplementary file 4 — Original western blots [file 41419_2025_7598_MOESM4_ESM.pdf]
